# Dynamic Metabolome and Transcriptome Profiling Provide Molecular Insights into Floral Bud Differentiation in *Michelia ‘Xin’*

**DOI:** 10.3390/biology14101383

**Published:** 2025-10-10

**Authors:** Yan Chen, Dapeng Li, Xiaoling Ji, Caixian Liu, Chenfei Huang

**Affiliations:** 1 College of Landscape Architecture, Central South University of Forestry and Technology, Changsha 410004, China; 2 School of Architecture and Art Design, Hunan University of Science and Technology, Xiangtan 411201, China; 3 Hunan Big Data Engineering Technology Research Center of Natural Protected Areas Landscape Resources, Changsha 410004, China

**Keywords:** *Michelia ‘Xin’*, flower bud, molecular pathway, metabolome profile, transcriptome dynamic, candidate genes

## Abstract

**Simple Summary:**

Floral bud differentiation (FBD) is a highly organized and regulated process crucial for the development of functional floral organs and plant reproduction. Despite the promising high ornamental value of *Michelia ‘Xin’*, scarce studies have been conducted on this species, limiting the understanding of its molecular functioning and improvement. This study aimed to characterize the phenology and molecular mechanisms underlying FBD in *Michelia ‘Xin’*. We characterized the FBD process into five stages (T1 to T5). FBD in *Michelia ‘Xin’* is stage-specifically regulated by synergy and interplay between phytohormones and transcription factors. The control of dormancy-related, flowering-promoting and circadian-related genes were essential for the induction and formation of reproductive organs. Key involved molecular pathways and candidate genes were screened out. Our findings offer important resources for dissecting the molecular network regulating FBD and improving the ornamental value of *Michelia ‘Xin’*.

**Abstract:**

*Michelia ‘Xin’* is an evergreen rare ornamental tree species that undergoes FBD only once but blooms twice a year. However, the molecular mechanisms controlling its FBD process remain largely unknown. This study characterized the FBD process and delved into the key molecular regulatory mechanisms through transcriptomic and metabolomic analyses of developing flower buds. FBD in *Michelia ‘Xin’* was characterized into five stages, including vegetative (T1), floral meristem transition (T2), tepal primordia differentiation (T3), stamen primordia differentiation (T4), and pistil primordia differentiation (T5). Analyses revealed a stage-specific metabolic and transcriptional regulation of FBD, with increasing numbers of differential metabolites and a decreasing number of DEGs from T1 to T5. Most phytohormone and transcription factor-related DEGs were highly induced from T2. The down-regulation of dormancy-associated protein homologs and CONSTANS-LIKE proteins associated with significant induction of flowering-promoting factor, CLAVATA3, trichome birefringence-like, and GRAVITROPIC IN THE LIGHT proteins was essential for the induction and reproductive organs’ development. Porphyrin biosynthesis, chlorophyll a-b binding proteins, DNA replication, flavonoid biosynthesis, and starch and sucrose metabolism were also significantly induced from T2. Key pivotal candidate genes were screened out. Our results provide fundamental resources for dissecting the molecular network regulating FBD and molecular-assisted flowering control in *Michelia ‘Xin’*.

## 1. Introduction

Flowering, the switch from vegetative to reproductive growth, is among the foremost essential developmental processes during angiosperm plants’ life cycle, ensuring successful progeny [1,2]. It is marked by FBD (flower bud differentiation), a well-organized and complex process involving genetic, epigenetic, and transcriptional reprogramming in response to particular temperatures and photoperiods [2,3,4,5]. During this process, the stem growth cone stops the production of axillary and leaf bud primordia to undergo a series of morphological, physiological, cytological, and histological changes to transform into floral organ primordia (sepal, petal, stamen, and pistil primordia) [6,7,8]. The floral development process is controlled by complex molecular networks encompassing specific hormones, genes, metabolites, and TFs (transcription factors) that incorporate environmental cues and endogenous signals for FBD and blooming to happen at the right time [9,10,11,12]. The regulation of FBD incorporates multilevel modulation of metabolite and gene expression levels in cells of floral organ primordia [11,13,14]. A wide diversity in the flowering process is observed between different plant types and species [13,14]. Therefore, a systematic dissection of the multilevel regulation of flowering processes in diverse plant species is a prerequisite for understanding flowering biology and optimizing ornamental plant breeding.

Tremendous studies on the model plant *Arabidopsis thaliana* revealed that the molecular regulation of floral transition in angiosperms primarily involves eight genetically interlinked pathways, including four endogenous pathways (autonomous, age-dependent, gibberellin, and trehalose-6-phosphate), four environmental pathways (photoperiod, vernalization, circadian clock, and temperature) [15,16]. The key flowering-related regulatory genes that control processes in these pathways have been uncovered, such as *FT* (FLOWERING LOCUS T), *CO* (CONSTANS), *SOC1* (SUPPRESSOR OF OVEREXPRESSION OF CONSTANS 1), *AP1* (APETALA1), *FLC* (FLOWERING LOCUS C), *GI* (GIGANTEA), *FBH* (FLOWERING bHLH), *ELF3* (EARLY FLOWERING 3), *FRI* (FRIGIDA), *COP1* (CONSTITUTIVE PHOTOMORPHOGENIC 1), etc. [5,16,17,18,19]. Each of these genes performs specific functions in regulating the floral process by interacting with other genes, phytohormones, and TFs at a transcriptional or protein level [20,21]. Plant hormones (gibberellins, jasmonic acid, salicylic acid, ethylene, cytokinin, auxins, brassinosteroids, and abscisic acid) and diverse TF family genes also play crucial regulatory roles in flowering through integrating signaling pathways and controlling the expression levels of flowering-related genes [13,22,23,24]. For instance, in Alfalfa, it was found that phytohormones GA, CK, IAA, and JA coordinately regulate the FBD process together with AP2/ERF, MADS-M-type, MYB, NAC, bHLH, WRKY, LFY, and HSF TF family genes [14].

*Michelia ‘Xin’* is an evergreen tree species in the Magnoliaceae family. It blooms twice a year in spring and summer, with a long flowering period, large flower volume, beautiful plant shape, and rich floral fragrance. It is an excellent ornamental tree species for gardens. Unlike annual plants, flowering regulation in woody species includes the control of floral bud initiation, dormancy, and bud development [25]. To illustrate, studies disclosed that the phenology and developmental process of floral transition and flower initiation in *Magnolia × soulangeana* ‘Changchun’, a species in the same plant family, are independent, with five FBD stages controlled mainly by DELLA-dependent gibberellin signaling [26,27]. In *Magnolia sinostellata*, FBD was also characterized into five stages, with MADS-box and AP2 family genes playing crucial regulatory roles [28]. Unfortunately, although *Michelia ‘Xin’* was discovered many years ago on the southern border as a natural hybrid (https://www.iplant.cn/info/Michelia%20cv.%20Xin (accessed on 21 May 2023)), it has not yet received in-depth research attention. Accordingly, the molecular regulation of FBD and flowering processes in *Michelia ‘Xin’* remains largely unknown. Therefore, it is of utmost importance to dissect the molecular regulatory network of FBD in *Michelia ‘Xin’*. Identifying the key molecular regulatory elements of the FBD process and understanding the involved mechanisms will help control the flowering time and improve its ornamental value.

The present study focuses on the dynamic molecular regulatory mechanisms controlling the FBD process in *Michelia ‘Xin’* through metabolite profiling and an in-depth analysis of the transcriptional expression patterns of key flowering-related genes. We aim to provide comprehensive molecular insights into the FBD process in *Michelia ‘Xin’* and fundamental resources for further functional studies and molecular-assisted flowering control.

## 2. Materials and Methods

### 2.1. Plant Materials and Growth Conditions

**Figure 1:**
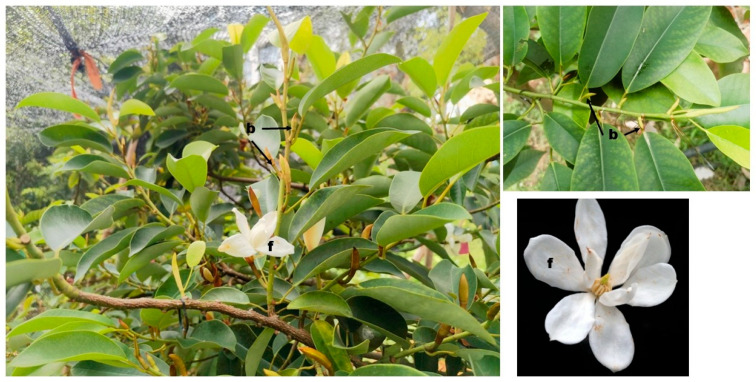
Photograph of *Michelia ‘Xin’* showing developing buds (b) and flower (f).

The *Michelia ‘Xin’* individuals (Figure 1) used for the present investigations are cultivated on the campus of Central South University of Forestry and Technology, Changsha, China (North latitude, between 27°51′ and 28°40′; East longitude, between 111°54′ and 114°15′). The school belongs to a typical subtropical humid climate. The average annual precipitation of 1361.6 mm is concentrated between April and July. The annual relative humidity is 80%, and the annual average temperature is 17.4 °C.

### 2.2. Morphological Observation

Three regularly maintained and managed 10-year-old pest and disease-free *Michelia ‘Xin’* trees (Figure 1) were selected as experimental materials. A total of three phenological observations were conducted from 2022 to 2024, following a previously reported method [27,28]. Briefly, floral transition occurs once each year from late April to early May in terminal buds. We randomly collected and observed fifteen buds from the middle parts of the trees every three days, starting from 28 March to 1 June. Samples were sliced (10 μm thickness) and stained in safranin fast green for 30 s. After bounding samples with neutral balata, they were observed and photographed (Axio Imager A2 microscope, Carl Zeiss, Oberkochen, Germany). Based on observations, fresh flower buds (seven) at five different stages, including vegetative (T1), floral meristem transition (T2), tepal primordia differentiation (T3), stamen primordia differentiation (T4), and pistil primordia differentiation (T5), were collected from six well-growing *Michelia ‘Xin’* trees in 2024. Samples were mixed to constitute one biological replicate. In total, we collected three biological replicates for each stage. Fresh samples were in situ frozen in liquid nitrogen and subsequently stored in a –80 °C freezer.

### 2.3. Metabolite Profiling of Developing Floral Buds

Freeze-dried samples (50 mg powder) were extracted with 70% pre-cooled (−20 °C) methanol (1.2 mL) by vortexing once every 30 min for 30 s, for a total of 6 times, followed by centrifugation (12,000× *g*, 8 min, and 4 °C) and supernatant collection. After filtration of extracts (0.22 μm micropore membrane filter, SCAA-104, Beijing Xin Cube Technology Development Co., Ltd., Beijing, China), LC-MS (Liquid chromatography-mass spectrometry) analysis was carried out using the QTRAP^®^ 6500+ mass spectrometer and the ExionLC™ AD system (SCIEX, Framingham, MA, USA) at Beijing Biomarker Technologies Co., LTD., Beijing, China, as per previously reported methods [29,30,31]. The qualitative identification of each metabolite was achieved by integrating spectrum, retention time (Rt), and mass spectra information. The Q1 (accurate precursor ions) value, Q3 (product ion) value, retention time, and fragmentation patterns were compared with standards (when they were available). When no standard was available, the metabolites were identified by reference to a local self-built database (Biomarker Technology Co., Ltd., Beijing, China) and public databases, including KNApSAcK (a comprehensive species-metabolite relationship database), MassBank (Europe high quality mass spectral database), HMDB (human metabolome database), METLIN (a metabolite mass spectral database), and MoTo DB (metabolome tomato database). All isotope signals were discarded to avoid duplication in the metabolite list [29,32]. Finally, we carefully checked all identified metabolites through comparison to the phytochemical dictionary (CRC, natural products database) and the literature. All identified metabolite’s relative content was computed via the MRM modes (QqQ MS analysis).

Metabolites with more than 20% missing values were excluded. A three-step process was then applied to remove redundant peaks, and the signal intensities were log-transformed. Multivariate analyses were conducted in R (v4.3.0). The packages pheatmap, MetaboAnalystR, and prcomp were, respectively, used for HCA (hierarchical clustering analysis), OPLS-DA (orthogonal partial least squares discriminant analysis), and PCA (principal component analysis). DMs (differential metabolites) were screened out by the ggplot2 program (R software, version 4.5.1) at thresholds of *p*-value < 0.05, |Log2FC| > 1, and VIP ≥ 1. Induced pathways were unveiled by KEGG analysis (http://www.kegg.jp/kegg/pathway.html, accessed on 16 May 2025).

### 2.4. Transcriptome Sequencing and Analysis

Total RNA extraction from bud samples was achieved by ethanol precipitation and CTAB-pBIOZOL [33]. Quality and quantity assessment of the RNA samples was carried out using a Qubit fluorescence quantifier and a Qsep400 high-throughput biofragment analyzer. The RNA sequencing was achieved on the Illumina HiSeq platform at Beijing Biomarker Technologies Co., LTD., Beijing, China, following previously described methods [34,35]. De novo assembly was conducted using Trinity v2.0.6 [36]. The assembly quality of transcripts was assessed using BUSCO [37]. We annotated all unigenes through BlastX (E-value > 1 × 10^−5^) against the Pfam (Protein family), Swiss Prot, Nr (NCBI non-redundant), Cluster of Orthologous Groups (COG/KOG), KEGG, eggnog, Gene Ontology (GO), and Trembl databases. Transcript normalization (calculation of FPKM values, fragments per kilobase per million mapped fragments) and abundance were achieved using Cufflinks (v2.2.1) [38]. DEGs detection was performed with the DESeq2 software [39], with criteria of FDR (false discovery rate) ˂ 0.05 and |Log2fold change| ≥ 1. GO and KEGG pathway enrichment analyses were carried out using Blast2GO [40] and KOBAS2.0 [41] programs, respectively. Significantly enriched terms/pathways were identified at a *p* or q value of ≤ 0.05. Microsoft Excel was used to organize the data. TBtools (v.2.224) was used to generate Venn diagrams and heatmaps [42].

### 2.5. RT-qPCR (Real-Time Quantitative Polymerase Chain Reaction)

Six transcripts were randomly selected for RT-qPCR analysis to confirm the reliability of the transcriptome data. First-strand cDNAs were synthesized using the TransScript^®^ First-Strand cDNA Synthesis SuperMix (TransGen Biotech, Beijing, China). RT-qPCR was conducted on the LightCycler96 instrument (Roche, Basel, Switzerland) with SYBR Green Mix (TIANGEN, Beijing, China). GAPDH (glyceraldehyde-3-phosphate dehydrogenase) gene served as the loading control [43]. The list of primers is presented in Table S8. Relative expression levels were determined using the 2^−ΔΔCt^ calculation method [44] in Excel.

## 3. Results

### 3.1. Morphological Changes During Flower Bud Differentiation in Michelia ‘Xin’

**Figure 2:**
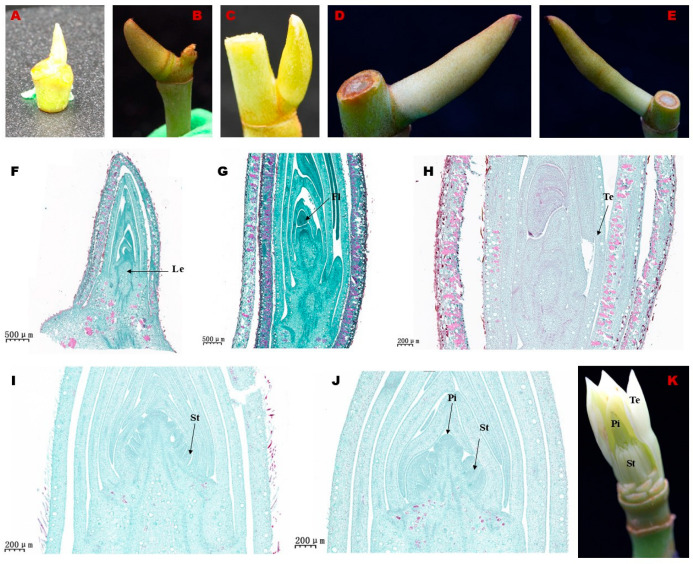
Morphological characteristics of the flower bud differentiation process in *Michelia ‘Xin’*. (**A**,**F**) Vegetative stage (T1). (**B**,**G**) Floral meristem transition stage (T2). (**C**,**H**) Tepal primordia differentiation stage (T3). (**D**,**I**) Stamen primordia differentiation stage (T4). (**E**,**J**) Pistil primordium differentiation stage (T5). (**K**) The fully developed flower bud. Le: leaf; Fl: Flower primordium; Te: Tetal primordium; St: Stamen primordium; Pi: Pistil base.

Based on the morphological features of FBD in *Michelia ‘Xin’*, we divided the process into five stages, including the vegetative stage (T1), floral meristem transition stage (T2), tepal primordia differentiation stage (T3), stamen primordia differentiation stage (T4), and pistil primordia differentiation stage (T5) (Figure 2). At T1, the buds were smooth and yellowish-green without scale hairs (Figure 2A). Closely arranged small differentiating primordium cells were observed (Figure 2F). At T2, the basal region of the bud started to enlarge, and yellowish brown hairs grew on the outer surface (Figure 2B). Inside developing buds, the spathe-like bracts launched into stratification and floral primordium became larger (Figure 2G). At T3, the bud grew wider and longer (Figure 2C). Inside, the tip of the growing floral meristem had an undulating surface, which indicates the initiation of tepal primordium differentiation (Figure 2H). During the T4 stage, buds increased in size, with denser outer yellowish-brown hairs (Figure 2D). The inner differentiation zone became larger, and multiple rows of small protruding spots (differentiating stamen primordia) developed at the bottom of the meristem (Figure 2I). At T5, the volume of buds increased further, and the outer hairs became downy (Figure 2E). The inner growing bud tip lengthened with a thicker base, and differentiating pistil primordia could be observed (Figure 2J). The complete differentiated bud is shown in Figure 2K.

### 3.2. Dynamic Metabolite Profile of Developing Flowers of Michelia ‘Xin’

**Figure 3:**
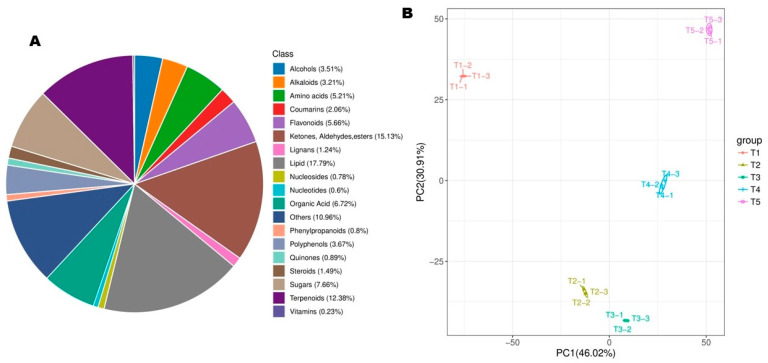
Metabolite profiles of developing floral buds at five different stages. (**A**) Classification of all identified metabolites. (**B**) Principal component analysis. T1, T2, T3, T4, and T5 indicate the vegetative stage, floral meristem transition stage, tepal primordia differentiation stage, stamen primordia differentiation stage, and pistil primordia differentiation stage, respectively.

To explore metabolome shifts and differential metabolic regulation occurring during FBD in *Michelia ‘Xin’*, flower bud samples at the five stages were subjected to LC-MS-based global metabolomics profiling. A total of 4361 metabolites were identified in the positive and negative ion modes in developing flower buds (Table S1). Lipids (17.79%), ketones, aldehydes and esters (15.13%), terpenoids (12.38%), sugars (7.66%), organic acids (6.72%), and flavonoids (5.66%) were the dominant metabolite classes (Figure 3A, Table S1). QC samples showed very high correlations of nearly 1, confirming the quality of the data and reproducibility of the experiment (Figure S1A).

Bud samples at T1, T2, T3, T4, and T5 exhibited considerable differences in metabolite profile and different patterns of metabolite accumulation as shown by the PCA and HCA (Figure 3B and Figure S1B). For instance, samples from different stages were completely separated on the PCA plot and could be distinguished by both PC1 (46.02%) and PC2 (30.91%) (Figure 3B). Most metabolites showed decreased accumulation patterns either from T1 or from T2 (Figure S1B). The observed significant metabolite profile differences of buds at the five stages were further supported by correlation analysis of samples (Figure S2). These results show that important metabolic shifts occurred during flower bud developmental transitions.

### 3.3. Differential Metabolites (DMs) and Pathways (DPs) Between Flowering Transition Stages

**Figure 4:**
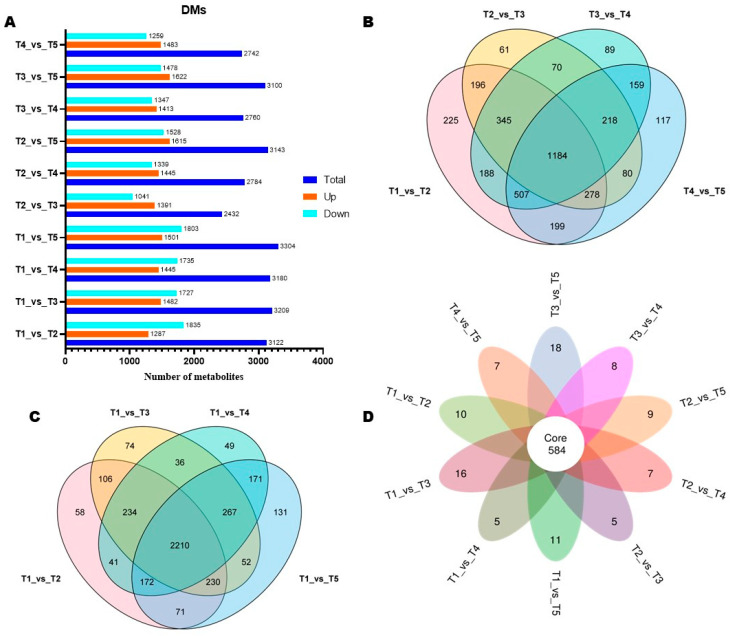
Differential metabolites (DMs). (**A**) Number of DMs in all pairwise comparisons. (**B**) Venn diagram of common DMs between T1_vs_T2, T2_vs_T3, T3_vs_T4, T4_vs_T5. (**C**) Venn diagram of overlapped DMs between T1_vs_T2, T1_vs_T3, T1_vs_T4, T1_vs_T5. (**D**) Petal map showing the number of core overlapped DMs. T1, T2, T3, T4, and T5 indicate the vegetative stage, floral meristem transition stage, tepal primordia differentiation stage, stamen primordia differentiation stage, and pistil primordia differentiation stage, respectively.

DMs offer the opportunity to identify differentially regulated pathways (DPs). We conducted an OPLS-DA analysis to identify DMs. The score plots of OPLS-DA supported the high difference in the metabolite profiles of flower buds of different stages (Figure S3). The OPLS-DA models showed strong goodness of fit and reliability, with an R^2^Y and Q^2^Y of 1 and ˃ 0.985, respectively (Figure S3A–D). We uncovered a total of 2432 to 3304 DMs in pairwise comparison between groups (Figure 4A). Notably, we detected 3122 (including 1287 up-regulated), 2432 (including 1391 up-regulated), 2760 (including 1413 up-regulated), and 2742 (including 1483 up-regulated) between T1_vs_T2, T2_vs_T3, T3_vs_T4, T4_vs_T5, respectively (Figure 4A and Figure S4A–D). Of them 1184 DMs were common to T1_vs_T2, T2_vs_T3, T3_vs_T4, T4_vs_T5(Figure 4B. When we considered T1 as the control group, we identified 2210 overlapped DMs (Figure 4C). The Petal diagram among DMs between all pairwise comparisons revealed a total of 584 core DMs (Figure 4D). The 584 core DMs are listed in Table S2.

**Figure 5:**
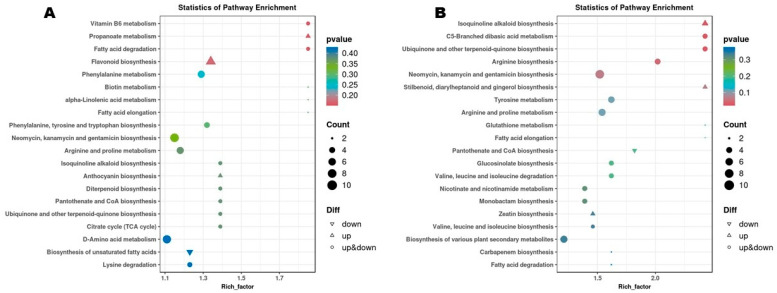
KEGG annotation and enrichment analysis of DMs between T1_Vs_T2 (**A**) and T2_Vs_T3 (**B**). T1, T2, and T3 indicate the vegetative stage, floral meristem transition stage, and tepal primordia differentiation stage, respectively.

We carried out KEGG analysis of DMs between T1_vs_T2, T2_vs_T3, T3_vs_T4, T4_vs_T5 to unveil dynamic DPs during flower bud developmental transitions. DMs between T1_Vs_T2 were primarily assigned to flavonoid biosynthesis, vitamin B6 metabolism, propanoate metabolism, fatty acid degradation, phenylalanine metabolism, neomycin, kanamycin and gentamicin biosynthesis, and arginine and proline metabolism (Figure 5A). Meanwhile, DMs between T2_Vs_T3 were mostly involved in neomycin, kanamycin and gentamicin biosynthesis, isoquinolin alkaloid biosynthesis, C5-branched dibasic acid metabolism, ubiquinone and other terpenoid biosynthesis, arginin biosynthesis, arginine and proline metabolism, and tyrosine metabolism (Figure 5B). Neomycin, kanamycin and gentamicin biosynthesis, porphyrin metabolism, flavonoid biosynthesis, and tyrosine metabolism were the major DPs between T3_Vs_T4 (Figure S5A). The major DPs between T4_Vs_T5 were porphyrin metabolism, isoflavonoid biosynthesis, arginine biosynthesis, biosynthesis of unsaturated fatty acid, flavonoid biosynthesis, flavone and flavonol biosynthesis, and phenylalanine metabolism (Figure S5B). Regarding the core DMs, they were mainly related to porphyrin metabolism, arginine and proline metabolism, and neomycin, kanamycin and gentamicin (Figure S6).

### 3.4. Dynamic Transcriptome Profile of Developing Flowers of Michelia ‘Xin’

**Figure 6:**
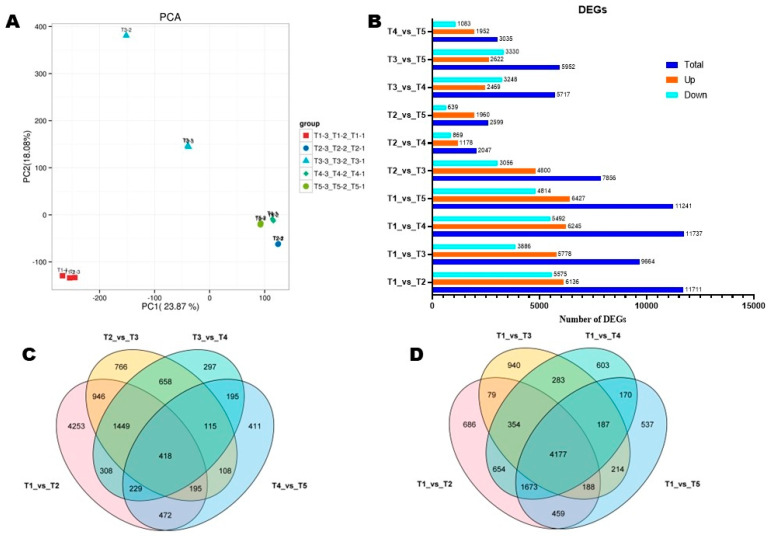
Transcriptome profiles of developing floral buds at five different stages. (**A**) Principal component analysis. (**B**) Number of DEGs (differentially expressed genes) in all pairwise comparisons. (**C**) Venn diagram of common DMs between T1_vs_T2, T2_vs_T3, T3_vs_T4, T4_vs_T5. (**D**) Venn diagram of overlapped DMs between T1_vs_T2, T1_vs_T3, T1_vs_T4, T1_vs_T5. T1, T2, T3, T4, and T5 indicate the vegetative stage, floral meristem transition stage, tepal primordia differentiation stage, stamen primordia differentiation stage, and pistil primordia differentiation stage, respectively.

To gain more molecular insights into the FBD process in *Michelia ‘Xin’*, bud samples collected at T1–T5 were subjected to RNA sequencing and analysis. The high-quality transcriptome sequencing yielded 18,014,166–27,252,017 bp of clean reads, with a GC content, Q20, and Q30 of all samples ranging from 45.31 to 47.23%, 99.79 to 99.93%, and 98.35 to 99.26%, respectively (Table S3). Using the Trinity software (v.2.15.1) [36,45], we de novo assembled clean reads into 99,937 unigenes, with an N50 value of 1496. The average length of transcripts and unigenes was 970.01 and 857.22, respectively (Figure S7). Sequence similarity analysis showed that 32.75% of unigenes are identical to sequences in *Cinnamomum micranthum* (Lauraceae) (Figure S8A). Consistent with the metabolite profiles, PCA and correlation analyses revealed that the transcriptome profiles of flower buds at different developmental stages were very different, indicating dynamic transcriptional regulation of the FBD process in *Michelia ‘Xin’* (Figure 6A and Figure S8B). Annotation to nine different databases indicated that 6.28, 26.95, 21.66, 16.65, 19.06, 19.76, 32.891, 27.41, and 32.45% unigenes were highly similar to known proteins in COG, GO, KEGG, KOG, Pfam, Swiss-Prot, Trembl, eggnog, and NR databases, respectively (Figure S8C). To confirm the reliability of the RNA-seq data, we randomly selected six unigenes for RT-qPCR analysis. As shown in Figure S9, the results by RT-qPCR and RNA-seq were consistent (r > 0.91).

### 3.5. Differentially Expressed Genes (DEGs) and Functional Annotation

The number of DEGs decreased along with the FBD process in *Michelia ‘Xin’* (Figure 6B). There were 11,711 (including 6136 up-regulated), 7856 (including 4800 up-regulated), 5717 (including 2469 up-regulated), and 3035 (including 1952 up-regulated) DEGs between T1_vs_T2, T2_vs_T3, T3_vs_T4, T4_vs_T5, respectively (Figure 6B). Only 418 DEGs were common between these four pairwise comparisons, suggesting different transcriptional regulation processes occurred at the five FBD stages (Figure 6C). When we considered T1 as the control group, we identified 4177 overlapped DEGs (Figure 6D).

**Figure 7:**
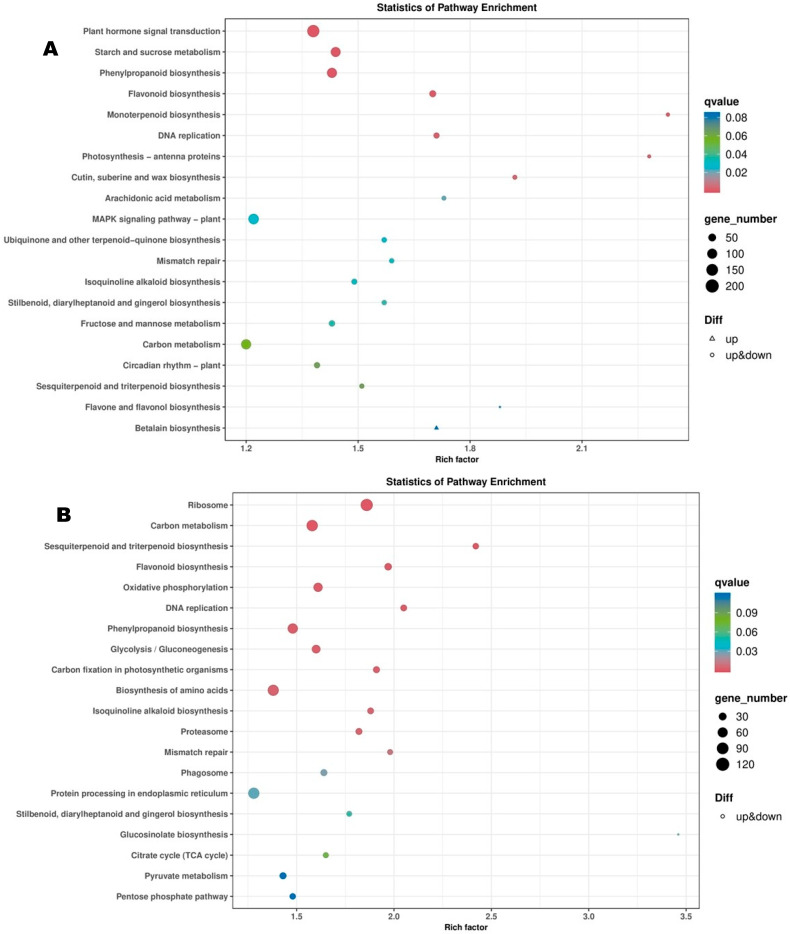
KEGG annotation and enrichment analysis of DEGs between T1_Vs_T2 (**A**) and T2_Vs_T3 (**B**). T1, T2, and T3 indicate the vegetative stage, floral meristem transition stage, and tepal primordia differentiation stage, respectively.

Functional annotation of DEGs to the KEGG pathways revealed that DEGs between T1_Vs_T2 were primarily involved in plant hormone signal transduction, starch and sucrose metabolism, phenylpropanoid biosynthesis, flavonoid biosynthesis, monoterpenoid biosynthesis, and DNA replication (Figure 7A). DEGs between T2_Vs_T3 were mostly involved in ribosome, carbon metabolism, sesquiterpenoid and triterpenoid biosynthesis, oxidation phosphorylation, flavonoid biosynthesis, phenylpropanoid biosynthesis, glycolysis/gluconeogenesis, biosynthesis of amino acids, and DNA replication (Figure 7B). Meanwhile, protein processing in endoplasmic reticulum, ribosome, DNA replication, oxidation phosphorylation, cutin, suberin and wax biosynthesis, and monoterpenoid biosynthesis were the major DPs between T3_Vs_T4 (Figure S10A). The major DPs between T4_Vs_T5 were plant hormone signal transduction, protein processing in endoplasmic reticulum, phenylpropanoid biosynthesis, flavonoid biosynthesis, starch and sucrose metabolism, monoterpenoid biosynthesis, cutin, suberin and wax biosynthesis, circadian rhythm, and cyanoamino acid metabolism (Figure S10B).

KEGG analysis of DEGs between T1_vs_T2, T1_vs_T3, T1_vs_T4, T1_vs_T5showed that plant hormone signal transduction, starch and sucrose metabolism, phenylpropanoid biosynthesis, MAPK signaling pathway, flavonoid biosynthesis, monoterpenoid biosynthesis, and DNA replication processes were pivotal for FBD in *Michelia ‘Xin’* (Figure 7A and Figure S11A–C). Supportively, GO analysis in the biological process assigned these DEGs mainly into “regulation of transcription, DNA templated”, “response to oxygen-containing compound”, “microtubule-based movement”, “response to hormone”, “response to endogenous stimulus”, “cell wall organization”, “response to chemical”, “response to lipid”, and “carbohydrate metabolic process” (Figure S12A–D).

### 3.6. Key Phytohormone-Related Genes Involved in FBD Process in Michelia ‘Xin’

**Figure 8:**
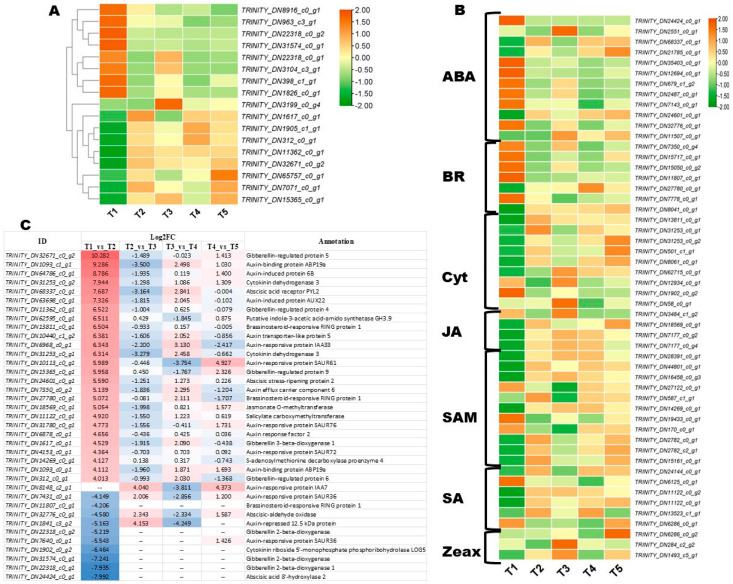
Differentially expressed phytohormone-related DEGs. (**A**) Expression patterns of gibberellin-related DEGs. (**B**) Expression patterns of other phytohormone-related DEGs, except auxin. (**C**) Expression fold changes of most differentially expressed phytohormone-related DEGs. The redder the color, the higher the fold changes, conversely to the blue color. T1, T2, T3, T4, and T5 indicate the vegetative stage, floral meristem transition stage, tepal primordia differentiation stage, stamen primordia differentiation stage, and pistil primordia differentiation stage, respectively.

Phytohormones are crucial for the coordinated initiation and smooth development of flowers. Therefore, we screened out phytohormone-related DEGs during FBD in *Michelia ‘Xin’*. In total, 153 phytohormone-related DEGs, including all types of plant hormones, were identified (Table S4). The expression patterns at the five FBD stages of gibberelline (GA), auxin, and other phytohormones are shown in Figure 8A,B and Figure S13, respectively. Most of these phytohormone-related DEGs exhibited stage-specific expression patterns. The GA genes *TRINITY_DN398_c1_g1*, *TRINITY_DN1826_c0_g1*, *TRINITY_DN963_c3_g1, TRINITY_DN22318_c0_g2, TRINITY_DN31574_c0_g1, TRINITY_DN22318_c0_g1, TRINITY_DN3104_c3_g1,* and *TRINITY_DN8916_c0_g1* were highly expressed only during the vegetative period (T1) (Figure 8A). Meanwhile, *TRINITY_DN3199_c0_g4* was highly expressed during tepal primordia differentiation (T3), while all other GA-related DEGs exhibited similar high expression from T2 to T5 (Figure 8A). Most ABA and BR (brassinosteroids)-related DEGs were also highly expressed during the vegetative period (T1) (Figure 8B). Three Jasmonoyl--L-amino acid synthetase genes (*TRINITY_DN7177_c0_g2*, JAR4; *TRINITY_DN7177_c0_g4*, JAR4; and *TRINITY_DN28391_c0_g1*; JAR6) exhibited the highest expression at T3 (tepal primordia differentiation) and T4 (stamen primordia differentiation) (Figure 8B).

To facilitate the selection of candidate genes, we filtered out most phytohormone-related DEGs (|Log2FC| > 4). The results revealed that significant induction of phytohormones was essential for the transition from the vegetative period (T1) to the floral meristem transition period (T2) (Figure 8C). The top 5 significantly induced genes during this transition include *TRINITY_DN32671_c0_g2* (gibberellin-regulated protein 5), *TRINITY_DN1093_c1_g1* (auxin-binding protein ABP19a), *TRINITY_DN64786_c0_g1* (auxin-induced protein 6B), *TRINITY_DN31253_c0_g2* (cytokinin dehydrogenase 3), and *TRINITY_DN68337_c0_g1* (abscisic acid receptor PYL2) (Figure 8C). *TRINITY_DN8148_c2_g1* (auxin-responsive protein IAA7) was highly induced during tepal primordia differentiation (T3) and pistil primordia differentiation (T5) (Figure 8C). *TRINITY_DN1841_c3_g2* (auxin-repressed 12.5 kDa protein) was highly induced during the vegetative period (T1) and tepal primordia differentiation (T3) (Figure 8C).

### 3.7. Key Transcription Factors (TFs) Regulating Flower Developmental Process in Michelia ‘Xin’

TFs are the master players in regulating plant growth and developmental processes. We identified a total of 549 DEGs encoding diverse TF family genes (Table S5). The top 20 most significantly induced TFs are presented in Table 1. As per the phytohormone-related DEGs, most significantly differentially regulated TFs (MSDR, |Log2FC| > 5) were highly expressed during the floral meristem transition period (T2), indicating that the interplay between TFs and phytohormone is crucial for flower bud induction in *Michelia ‘Xin’* (Table S5). For instance, 49 out of the 78 MSDR TFs were highly expressed at T2, with the top five genes being *TRINITY_DN1305_c5_g1* (MADS9), *TRINITY_DN2535_c0_g1* (B3 domain-containing protein), *TRINITY_DN9192_c0_g1* (MADS-box transcription factor 6), *TRINITY_DN30055_c0_g3* (NAC domain-containing protein 104), and *TRINITY_DN18152_c0_g1* (Zinc finger CCCH domain-containing protein 9) (Table 1 and Table S5). *TRINITY_DN22896_c0_g1* (Myb-related protein 340), *TRINITY_DN2045_c7_g1* (NAC transcription factor 47), and *TRINITY_DN163_c1_g1* (ERF110) were highly induced during pistil primordia differentiation (T5) (Table 1 and Table S5).

### 3.8. Circadian and Flowering-Related DEGs

**Figure 9:**
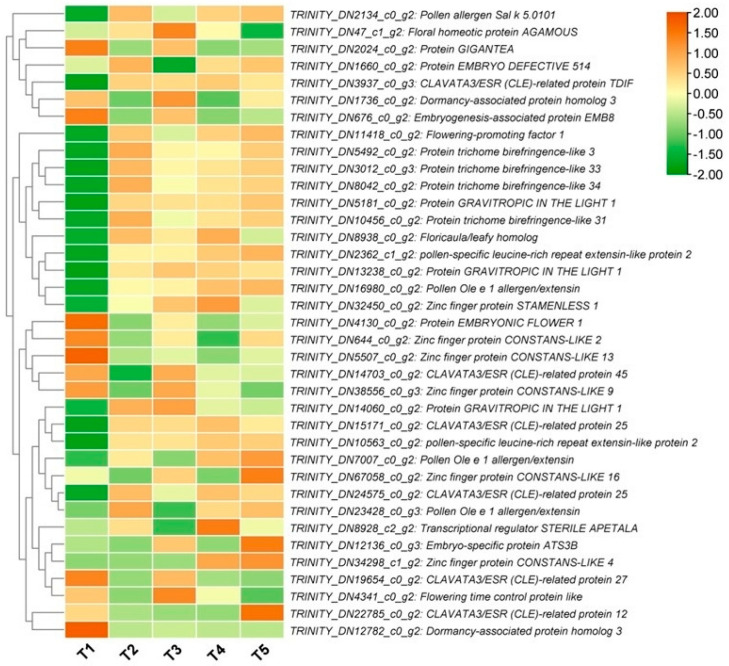
Expression patterns of flowering and circadian/photoperiod-related DEGs. T1, T2, T3, T4, and T5 indicate the vegetative stage, floral meristem transition stage, tepal primordia differentiation stage, stamen primordia differentiation stage, and pistil primordia differentiation stage, respectively.

The transition from vegetative growth to reproductive development is mediated by the photoperiodic response to sensing the length of night/day through circadian regulation of light-signaling molecules [46,47]. Therefore, to better understand the FBD process in *Michelia ‘Xin’*, we screened out differentially expressed circadian and flowering-related DEGs. In total, 37 genes were identified (Table S6), and their expression patterns are shown in Figure 9. Most of them showed high expression from T2 (floral meristem transition period) to T5 (pistil primordia differentiation stage) compared to T1 (vegetative period) (Figure 9). Four protein trichome birefringence-like genes (*TRINITY_DN5492_c0_g1*, *TRINITY_DN10456_c0_g1*, *TRINITY_DN3012_c0_g2*, and *TRINITY_DN8042_c0_g1*) were up-regulated more than 5.6-fold during the floral meristem transition period (T2) (Table S6). Floricaula/leafy homolog (*TRINITY_DN8938_c0_g1*) and flowering-promoting factor 1 (*TRINITY_DN11418_c0_g1*) were also up-regulated during the floral meristem transition period (T2) by 4.302-fold and 11.729-fold, respectively (Table S6). Transcriptional regulator STERILE APETALA (*TRINITY_DN8928_c2_g1*) and CLAVATA3/ESR (CLE)-related protein 12 (*TRINITY_DN22785_c0_g1*) were up-regulated over 5.2-fold at T4 (stamen primordia differentiation stage) and T5 (pistil primordia differentiation stage), respectively (Table S6). Two dormancy-associated protein homolog 3 genes (*TRINITY_DN12782_c0_g1* and *TRINITY_DN1736_c0_g1*), one protein EMBRYONIC FLOWER 1 gene (*TRINITY_DN4130_c0_g1*), and three Zinc finger protein CONSTANS-LIKE genes (*TRINITY_DN5507_c0_g1*, *TRINITY_DN644_c0_g1*, and *TRINITY_DN38556_c0_g2*) were significantly down-regulated during the transition from T1 (vegetative period) to T2 (floral meristem transition period) (Figure 9, Table S6). Meanwhile, one Zinc finger protein STAMENLESS 1 gene (*TRINITY_DN32450_c0_g1*) and three protein GRAVITROPIC IN THE LIGHT 1 genes (*TRINITY_DN14060_c0_g1, TRINITY_DN13238_c0_g1*, and *TRINITY_DN5181_c0_g1*) were up-regulated over 2.4-fold during the transition from T1 to T2 (Figure 9, Table S6).

In addition, as energy supply is essential for reproductive organs’ development, we examined the expression patterns of chlorophyll-related DEGs. The results showed that most chlorophyll a-b binding protein-related DEGs were induced from T2 (floral meristem transition period) to T5 (pistil primordia differentiation stage) compared to T1 (vegetative period) (Figure S14, Table S7).

## 4. Discussion

FBD is the first step of plant reproductive development, and normal completion of this process determines the successful reproduction of angiosperm plants via seeds [8]. *Michelia ‘Xin’* is an important woody ornamental plant. This study characterized the FBD process in *Michelia ‘Xin’* and unveiled key regulatory genes, metabolites, and pathways through combined transcriptomics and metabolomics analysis of developing buds. We found that the FBD process in *Michelia ‘Xin’* is separated into five stages, similar to reports in *Magnolia × soulangeana* ‘Changchun’ and *Magnolia sinostellata* [27,28]. These results highlight the similarity of FBD processes in members of the Magnoliaceae family. The dynamic transcriptome and metabolome profiling revealed a stage-specific regulation of the FBD process in *Michelia ‘Xin’*, with the main controlled molecular processes, including plant hormone signal transduction, phenylpropanoid biosynthesis, starch and sucrose metabolism, MAPK signaling pathway, flavonoid biosynthesis, monoterpenoid biosynthesis, and DNA replication. Further studies should dissect the molecular network regulating these processes during floral development in *Michelia ‘Xin’* and other woody plants.

FBD in angiosperms is a response to various endogenous and exogenous cues that result in flowering [48,49]. The switch from vegetative to reproductive growth is primarily underlined by the ambient temperature and photoperiod [5,16,50]. Most photoperiod/light/circadian-related DEGs were induced from T2. This result indicates that FBD in *Michelia ‘Xin’* is a response to photoperiod and ambient temperature signals. Zinc finger TFs encoding *CO* play critical roles in regulating FBD in response to photoperiod-induced molecular signals [51]. A prolonged photoperiod triggers higher expression of *CO*, which subsequently activates *SOC1* and *FT*, the master regulators and promoters of flowering [52]. *GI* positively controls the expression of *CO* and *FT* [16,17,53]. However, the molecular functions of *CO* differ from those of *COL* (CO-like) genes [54]. For example, *AtCOL4* and *AtCOL9* in *Arabidopsis* act as transcriptional inhibitors of flowering by controlling the flowering time [55,56]. *OsCOL15* suppresses flowering in rice by activating *RID1* and *Ghd7* [57]. Here, we found that two dormancy-associated protein homolog 3 genes (*TRINITY_DN12782_c0_g1* and *TRINITY_DN1736_c0_g1*), one protein EMBRYONIC FLOWER 1 gene (*TRINITY_DN4130_c0_g1*), and three *COL* genes (*TRINITY_DN5507_c0_g1*, *TRINITY_DN644_c0_g1*, and *TRINITY_DN38556_c0_g2*) were significantly down-regulated during the transition from vegetative to floral meristem transition period. These genes might cooperate to inhibit FBD and repress flowering from taking place before favorable environmental conditions are reunited. Two genes, *TRINITY_DN8938_c0_g1* and *TRINITY_DN11418_c0_g1,* that encode floricaula/leafy homolog and flowering-promoting factor 1 were up-regulated from the floral meristem transition stage. These genes may be the key stimulators and regulators of FBD and flowering in *Michelia ‘Xin’*. *FPF1* is a key player in the GA pathway, regulating the floral induction in apical meristems [58,59]. *LFY* (LEAFY) is associated with floral identity and initiates the transition from vegetative to floral meristem [60]. Other flowering-related genes, such as *SAP* (STERILE APETALA, *TRINITY_DN8928_c2_g1*) and some *CLE* (CLAVATA3/ESR), which exhibited up-regulated expression at certain points between T2 and T5, may also play critical regulatory functions during FBD and the flowering processes in *Michelia ‘Xin’*. *SAP* is essential for maintaining floral identity, normal floral organogenesis, and normal development of inflorescence and flower [61,62]. Collectively, these results show that the FBD process in *Michelia ‘Xin’* is regulated by both endogenous and environmental signals. In support, Nie et al. found that the contrasting fertility between spring (fertile) and summer (sterile) flower buds in *Magnolia × soulangeana ‘Changchun’* is caused by differences in phytohormone levels, differential transcriptional regulations, and environmental temperature variation [63].

Functional analysis of DEGs highlighted that plant hormone signal transduction and MAPK signaling are the prime regulatory mechanisms of FBD in *Michelia ‘Xin’*, with phytohormones and TFs playing crucial roles. Most of the DEGs encoding TFs and phytohormones were induced from the T2 stage. These results align with reports in grape, broccoli, *Camellia oleifera*, and Alfalfa [13,14,48,64]. The roles of TFs in flower initiation and development have been explored in many plant species [16,65,66,67]. The top five highly induced TFs included *MADS9* (*TRINITY_DN1305_c5_g1*), B3 domain-containing protein (*TRINITY_DN2535_c0_g1*), *MADS6* (*TRINITY_DN9192_c0_g1*), *NAC104* (*TRINITY_DN30055_c0_g3*), and Zinc finger *CCCH9* (*TRINITY_DN18152_c0_g1*). It is well documented that MADS-box TF family genes are highly expressed at the early stages of FBD to promote early flowering through interactions with phytohormones and other TFs [28,68,69,70]. Sun et al. uncovered that DELLA-dependent GA-mediated signaling cascades control floral transition in *Magnolia* × *soulangeana ‘Changchun’* [27]. These findings suggest that DELLA proteins may play central regulatory roles during FBD in *Michelia ‘Xin’* through their multilevel interactions with GA [71,72]. In this study, we found that almost all phytohormone-related family genes were induced during the FBD from stage T2, indicating that all hormone types participate in the regulation networks and processes. The diverse regulatory functions of phytohormones during reproductive developmental processes in plants have been extensively studied [1,15,22,23]. Taken together, these findings show that the FBD in *Michelia ‘Xin’* is coordinately regulated by the interplay between TFs, phytohormones, and flowering-related genes.

Gui et al. revealed that JA and cytokinin interact with MADS TF family genes to regulate flower development in *Zanthoxylum armatum* [69]. Reports indicate that JA plays crucial functions in plant reproduction, including sex determination, male fertility, and seed maturation [23]. Many metabolites, such as neomycin, propyl gallate, lyn3, etc., can effectively inhibit JA-induced reactions in plants [73,74,75]. The metabolomics results showed that neomycin, kanamycin and gentamicin biosynthesis were specifically controlled during the FBD in *Michelia ‘Xin’*. Notably, flavonoid biosynthesis and associated metabolic pathways, such as phenylalanine metabolism and tyrosine metabolism, were induced from stage T2. In addition, the identified highly induced TFs are known to coordinately regulate flavonoid biosynthesis [68,76]. These results suggest they may play key regulatory functions during FBD in *Michelia ‘Xin’*. Flavonoid substances were found to play a pivotal role in flower development in plants [76,77]. For instance, in *Juglans sigillata* Dode, flavonoid biosynthesis was identified as a key pathway influencing female FBD, with metabolites primarily shifting towards the isoflavonoid, flavone, and flavonol branches, and structural genes modulating key flowering regulatory genes including *SOC1*, Constans, Flowering Locus T, and APETALA1 [68]. Furthermore, we identified 584 key DMs, including sugars, lipids, amino acids, etc. Many carbohydrates (sucrose, fructans, starch, cellulose, hemicellulose, trehalose-6-phosphate, etc.), lipids, amino acids, polyamines, and phenolics have been proven to involve in the regulation of floral organ initiation and development [78]. These findings highlight the importance of metabolic adjustment and modulation during FBD in *Michelia ‘Xin’*. Future studies should screen and validate flowering-promoting and flowering-inhibitory metabolites for exogenous control of flowering time and flower production in *Michelia ‘Xin’*. The interplay of regulatory roles between metabolites, phytohormones, TFs, and flowering-related genes should be paid particular attention to.

A previous study revealed that FBD requires starch accumulation while consuming soluble sugars [79]. Consistent with this, we found that starch and sucrose metabolism and amino acid metabolism were induced during the FBD from T2 in *Michelia ‘Xin’*. The metabolism of photosynthesis-related elements (porphyrin biosynthesis and expression levels of chlorophyll a-b binding proteins) was also significantly induced from T2. These findings show that enhanced energy production and supply are essential for normal FBD in *Michelia ‘Xin’*. *GI* exhibited high expression levels during T1 and T3, inferring that it may also play critical regulatory functions during FBD in *Michelia ‘Xin’*. *GI* is a plant-specific nuclear protein that plays key roles in diverse physiological processes, such as light signaling, control of flowering time and circadian rhythm, FBD, hypocotyl elongation, sucrose signaling, stress tolerance, starch accumulation, chlorophyll accumulation, etc. [17]. We unveiled several potential candidate genes, including phytohormones, TFs, and circadian/flowering-related genes. These genes must be functionally characterized to deepen our knowledge of the complex regulatory network of FBD in woody plants and pave the way for the molecular-assisted production of desirable *Michelia ‘Xin’* flowers.

## 5. Conclusions

Collectively, this study characterizes the FBD process in *Michelia ‘Xin’* and shows that the five stages are specifically controlled by a coordinated interaction between phytohormone and TFs, with them being crucial for the transition from vegetative (T1) to floral meristem transition (T2). The down-regulation of dormancy-related genes coupled with high induction of key flowering-promoting and circadian-related genes was also essential for the transition from T1 to T2 and the differentiation of flower organs. Porphyrin biosynthesis, chlorophyll a-b binding proteins, and starch and sucrose metabolism were significantly induced during reproductive tissues’ differentiation, highlighting the importance of enhanced energy production for normal FBD in *Michelia ‘Xin’*. In addition, enhanced DNA replication, flavonoid biosynthesis, arginine and proline metabolism, and tyrosine metabolism, and the control of neomycin, kanamycin and gentamicin biosynthesis were also pivotal for the differentiation of flower tissues in *Michelia ‘Xin’*. Our findings offer an insightful molecular understanding of the FBD process in *Michelia ‘Xin’*. Moreover, they provide valuable resources for deciphering the molecular network regulating the process and for molecular-assisted improvement of the horticultural value of *Michelia ‘Xin’*.

## Figures and Tables

**Table 1:** List of the top 20 significantly induced transcription factors during flower bud development in *Michelia ‘Xin’.* The redder the color, the higher the fold changes, conversely to the blue color.

ID	Log2FC				Annotation
	T1_vs_T2	T2_vs_T3	T3_vs_T4	T4_vs_T5	
*TRINITY_DN1305_c5_g1*	11.958	1.479	0.277	0.148	Agamous-like MADS-box protein MADS9
*TRINITY_DN2535_c0_g1*	11.146	−1.720	1.971	−0.600	B3 domain-containing protein
*TRINITY_DN9192_c0_g1*	11.104	0.368	0.463	0.071	MADS-box transcription factor 6
*TRINITY_DN30055_c0_g3*	9.955	−1.088	0.671	0.862	NAC domain-containing protein 104
*TRINITY_DN18152_c0_g1*	9.801	−1.859	0.856	0.101	Zinc finger CCCH domain-containing protein 9
*TRINITY_DN6301_c0_g1*	9.647	2.300	0.135	0.398	Agamous-like MADS-box protein MADS4
*TRINITY_DN4827_c0_g1*	9.043	−2.068	2.325	−0.243	B3 domain-containing protein Os06g0194400
*TRINITY_DN11681_c0_g1*	9.042	−0.641	−0.207	−0.238	Transcription factor WER
*TRINITY_DN604_c3_g2*	8.766	−4.143	4.401	−3.295	Transcription factor TRY
*TRINITY_DN17178_c0_g1*	8.650	−3.142	3.312	−1.467	Transcription factor bHLH96
*TRINITY_DN3413_c4_g2*	8.574	−0.188	0.042	0.049	MADS-box transcription factor 17
*TRINITY_DN16_c1_g1*	8.535	−1.442	0.530	1.226	Transcription factor bHLH146
*TRINITY_DN8450_c0_g1*	8.394	−0.919	0.522	1.240	Transcription factor PAR2
*TRINITY_DN4446_c0_g1*	8.262	−0.710	0.924	0.070	F-box protein At1g10780
*TRINITY_DN22896_c0_g1*	--	--	--	8.385	Myb-related protein 340
*TRINITY_DN65996_c0_g1*	7.989	−0.753	−1.526	--	WAT1-related protein At1g21890
*TRINITY_DN203_c0_g1*	7.825	−3.287	1.255	2.045	Transcription factor MYB1
*TRINITY_DN10061_c0_g1*	7.688	−2.343	3.145	−0.731	Basic leucine zipper 34
*TRINITY_DN7028_c0_g1*	7.373	−3.025	--	1.794	Transcription factor MYB1
*TRINITY_DN2070_c1_g2*	7.296	−1.582	−0.365	1.891	Transcription factor MYB83

## Data Availability

The raw RNA-seq data are available as SRAs (accession ID, PRJNA1269252) from NCBI at https://www.ncbi.nlm.nih.gov/bioproject/?term=PRJNA1269252 (accessed on 21 May 2023). All other datasets generated and/or analyzed during the current study are available from the corresponding author upon reasonable request.

## Supplementary Materials

The following supporting information can be downloaded at: https://www.mdpi.com/article/10.3390/biology14101383/s1. Figure S1: Correlation analysis of QC and HCA analysis; Figure S2. Correlation analysis of all other samples; Figure S3: OPLS-DA plot of DEGs between T1_vs_T2, T2_vs_T3, T3_vs_T4, T4_vs_T5, respectively; Figure S4: Volcano plot of DMs; Figure S5: KEGG annotation and enrichment analysis of DMs between T3_Vs_T4 (**A**) and T4_Vs_T5 (**B**); Figure S6: KEGG annotation and enrichment analysis of core overlapped DMs; Figure S7: Distribution of the length of Unigenes and transcripts; Figure S8: Homology analysis results of Unigenes and Correlation analysis of samples based on the transcriptome data; Figure S9: qRT-PRC validation of the RNA-seq data; Figure S10: KEGG annotation and enrichment analysis of DEGs between T3_vs_T4 and T4_vs_T5; Figure S11: KEGG annotation and enrichment analysis of DEGs between T1_vs_T2, T1_vs_T3, T1_vs_T4, T1_vs_T5, respectively; Figure S12: GO annotation and enrichment analysis of DEGs between T1_vs_T2, T1_vs_T3, T1_vs_T4, T1_vs_T5, respectively; Figure S13: Expression patterns of auxin-related DEGs; Figure S14: Expression patterns of Chlorophyll-related DEGs; Table S1: List of all identified metabolites in developing floral buds; Table S2: List of core overlapped differential metabolites; Table S3: Summary of the high-quality RNA-seq data; Table S4: List of phytohormone-related DEGs; Table S5: List of differentially expressed transcription factors; Table S6: List of flowering and circadian/photoperiod-related DEGs; Table S7: List of chlorophyll-related DEGs; Table S8: List of primers used for qRT-PCR analysis.
