# Supplementary material for: Dynamic Metabolome and Transcriptome Profiling Provide Molecular Insights into Floral Bud Differentiation in Michelia ‘Xin’
Source: Biology (Basel). 2025 Oct 10;14(10):1383. doi: 10.3390/biology14101383 (PMC12561709; doi:10.3390/biology14101383)
Supplement: Supplementary file 1 [file biology-14-01383-s001.zip › biology-3894934-supplementary/Supplementary Figures.pdf]

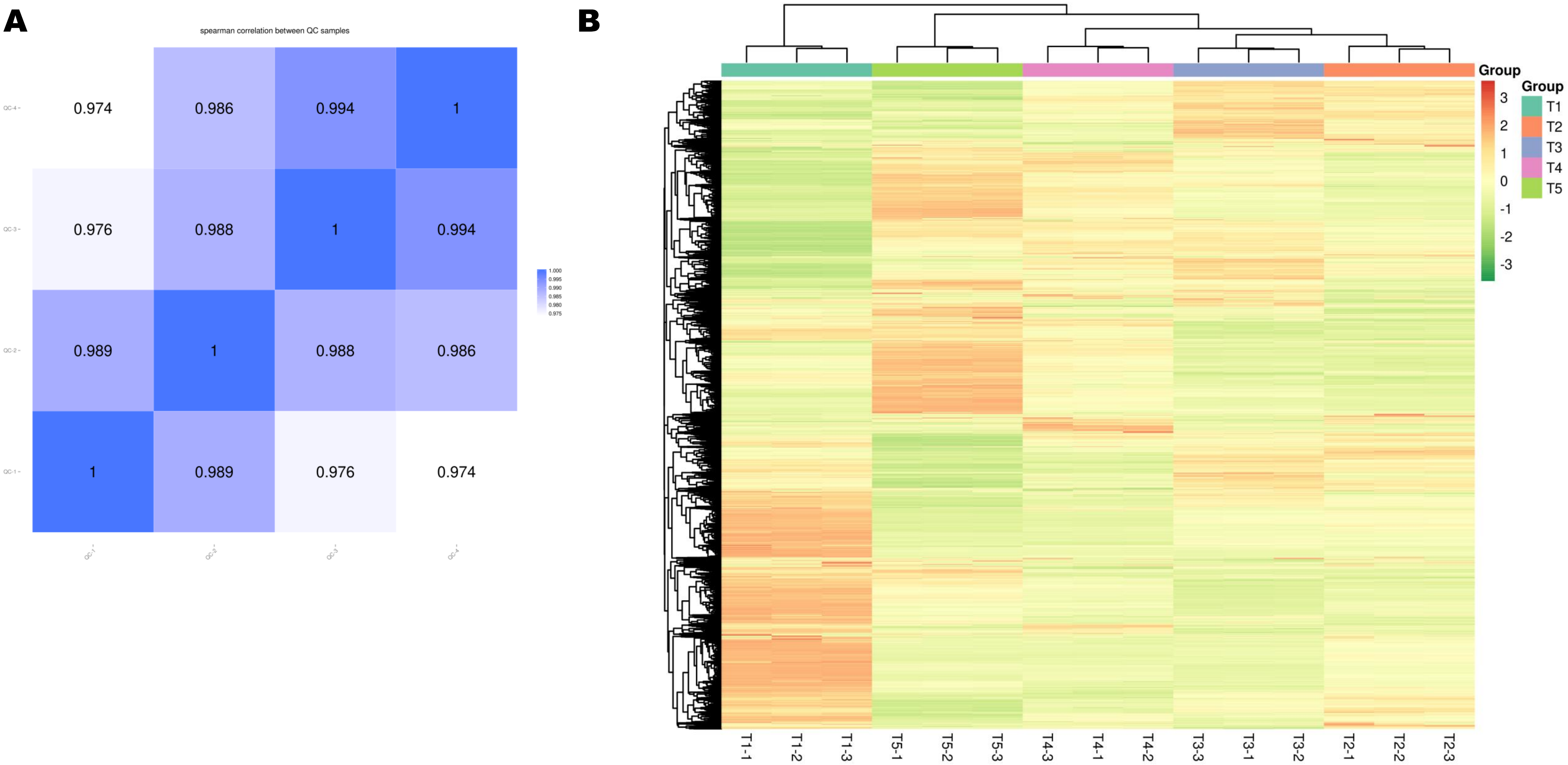

Figure S1. (A) Correlation analysis of QC samples. (B) Hierarchical clustering analysis of metabolite profiles of developing floral buds at five different stages. T1, T2, T3, T4, and T5 indicate the vegetative stage, floral meristem transition stage, tepal primordia differentiation stage, stamen primordia differentiation stage, and pistil primordia differentiation stage, respectively.

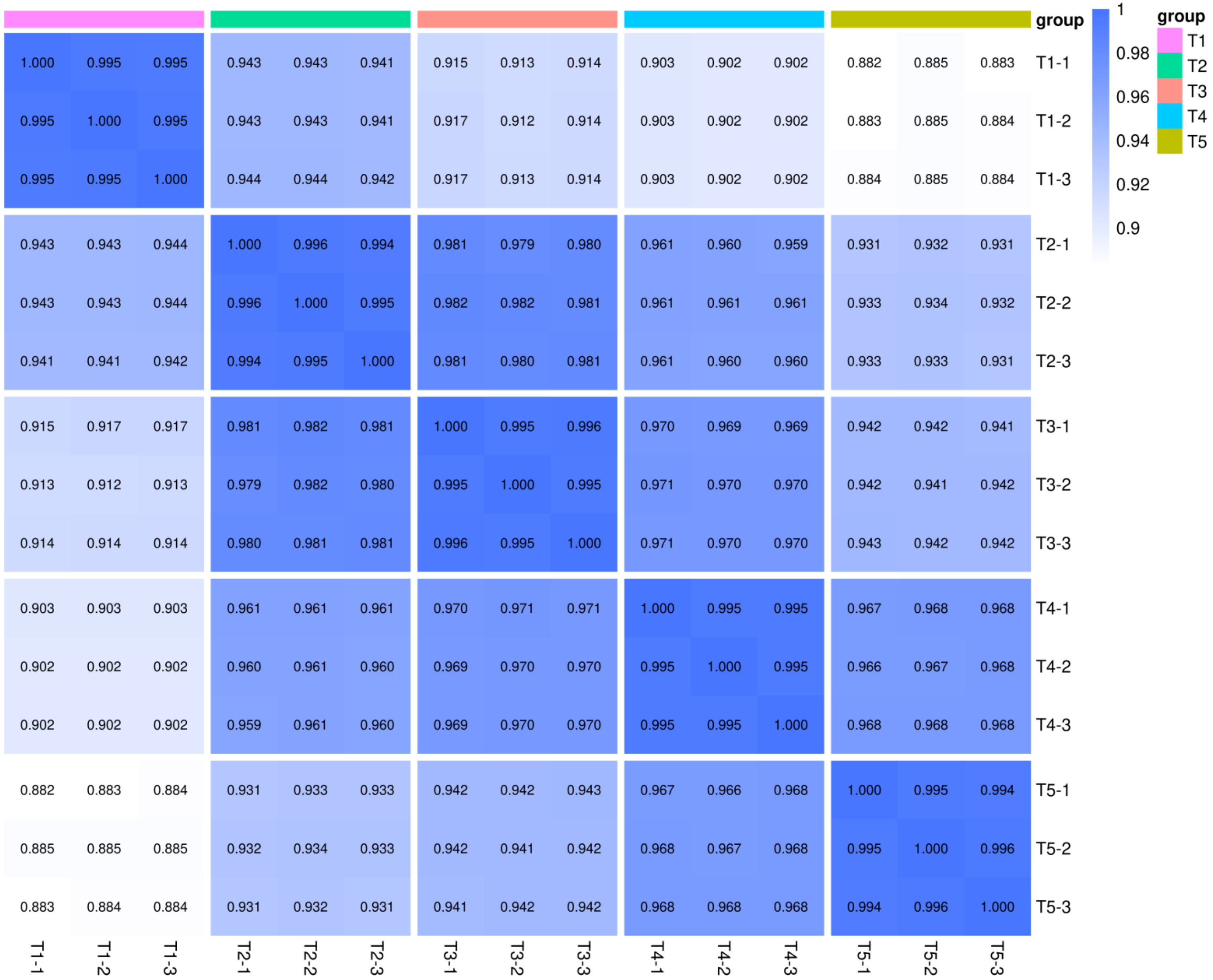

Figure S2. Correlation analysis of samples (B). T1, T2, T3, T4, and T5 indicate the vegetative stage, floral meristem transition stage, tepal primordia differentiation stage, stamen primordia differentiation stage, and pistil primordia differentiation stage, respectively.

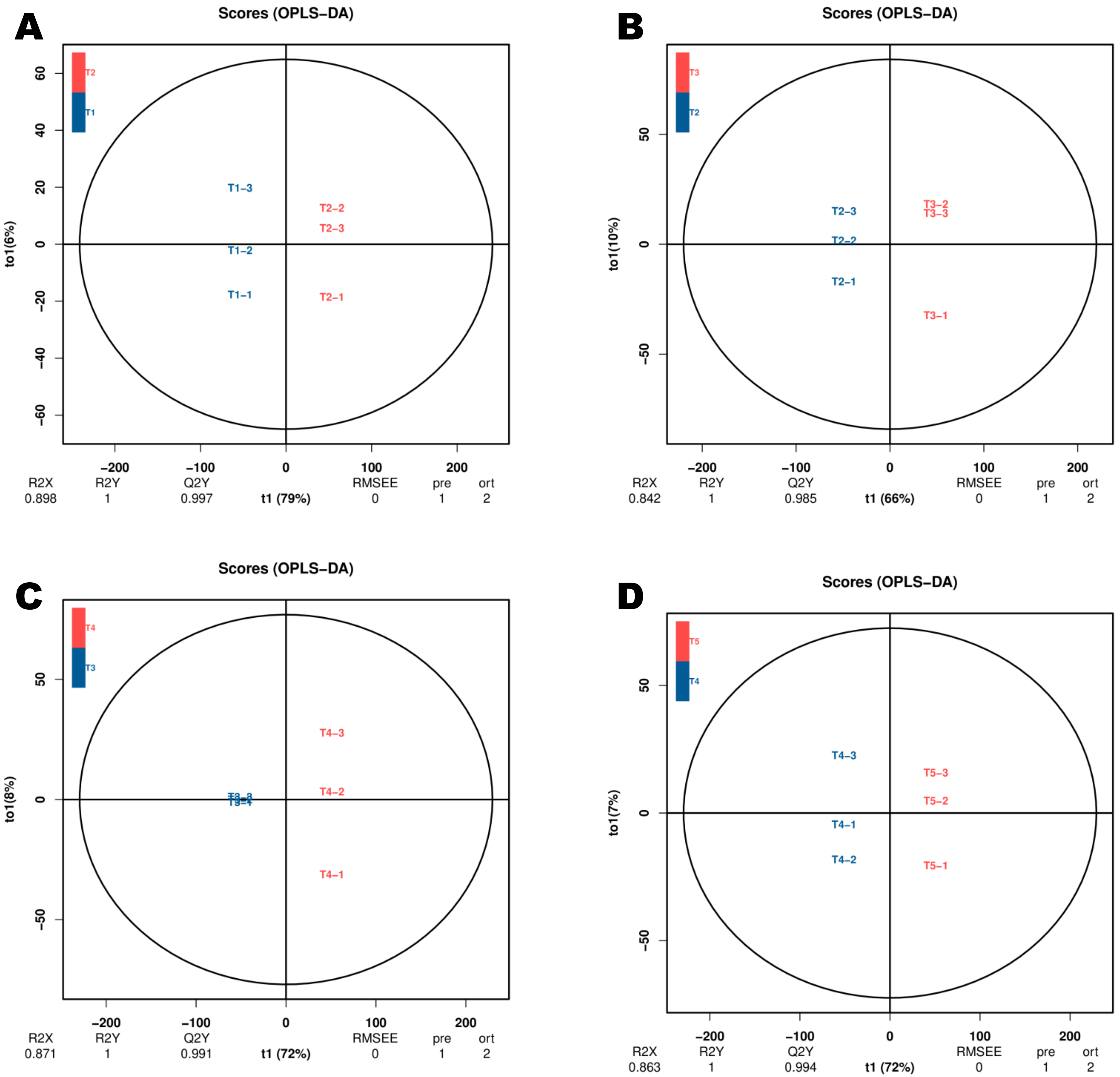

Figure S3. A- D) OPLS-DA plot of DEGs between T1_vs_T2, T2_vs_T3, T3_vs_T4, T4_vs_T5, respectively. T1, T2, T3, T4, and T5 indicate the vegetative stage, floral meristem transition stage, tepal primordia differentiation stage, stamen primordia differentiation stage, and pistil primordia differentiation stage, respectively.

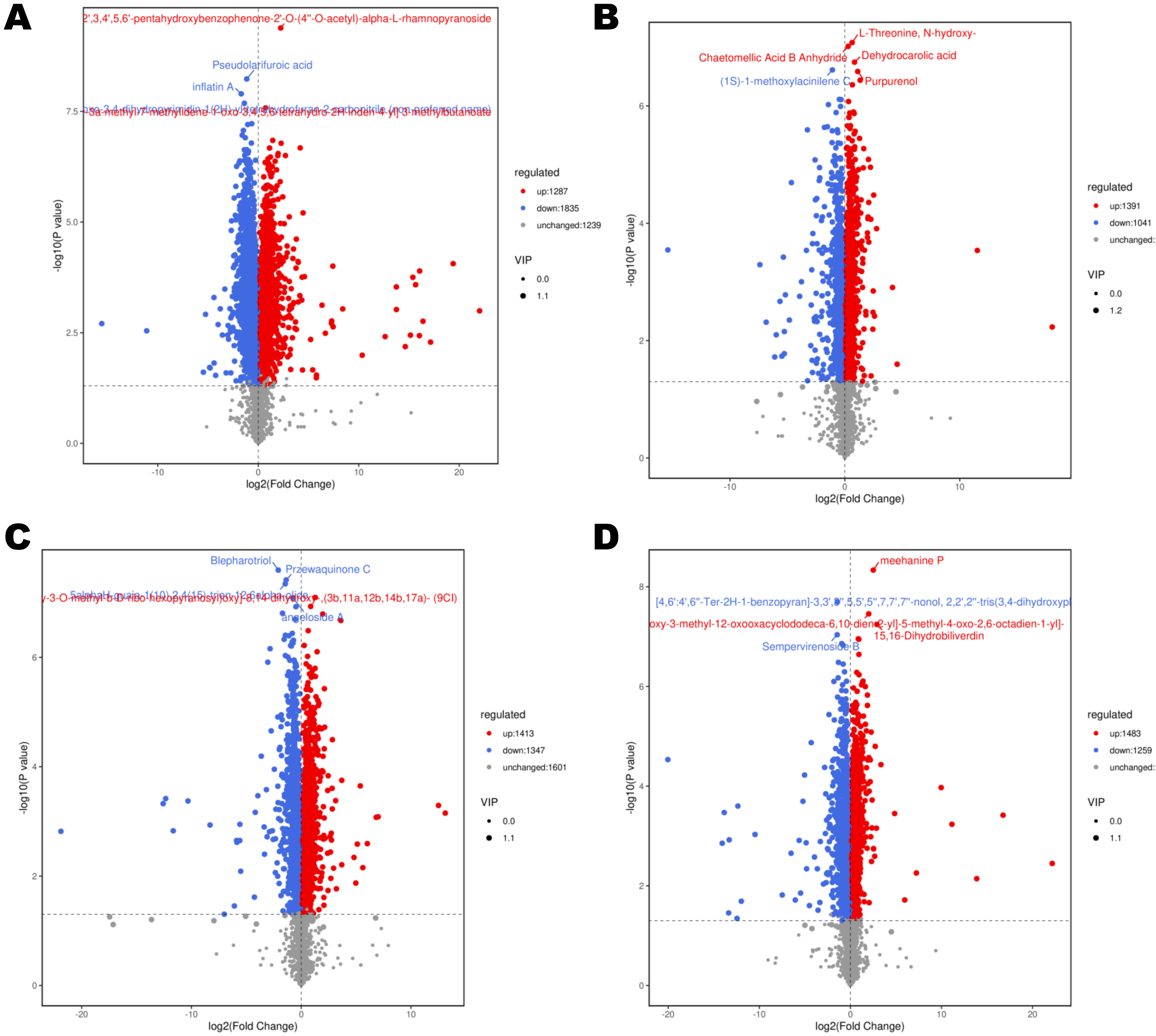

Figure S4. Volcano plot of DMs between T1_vs_T2, T2_vs_T3, T3_vs_T4, T4_vs_T5, respectively. T1, T2, T3, T4, and T5 indicate the vegetative stage, floral meristem transition stage, tepal primordia differentiation stage, stamen primordia differentiation stage, and pistil primordia differentiation stage, respectively.

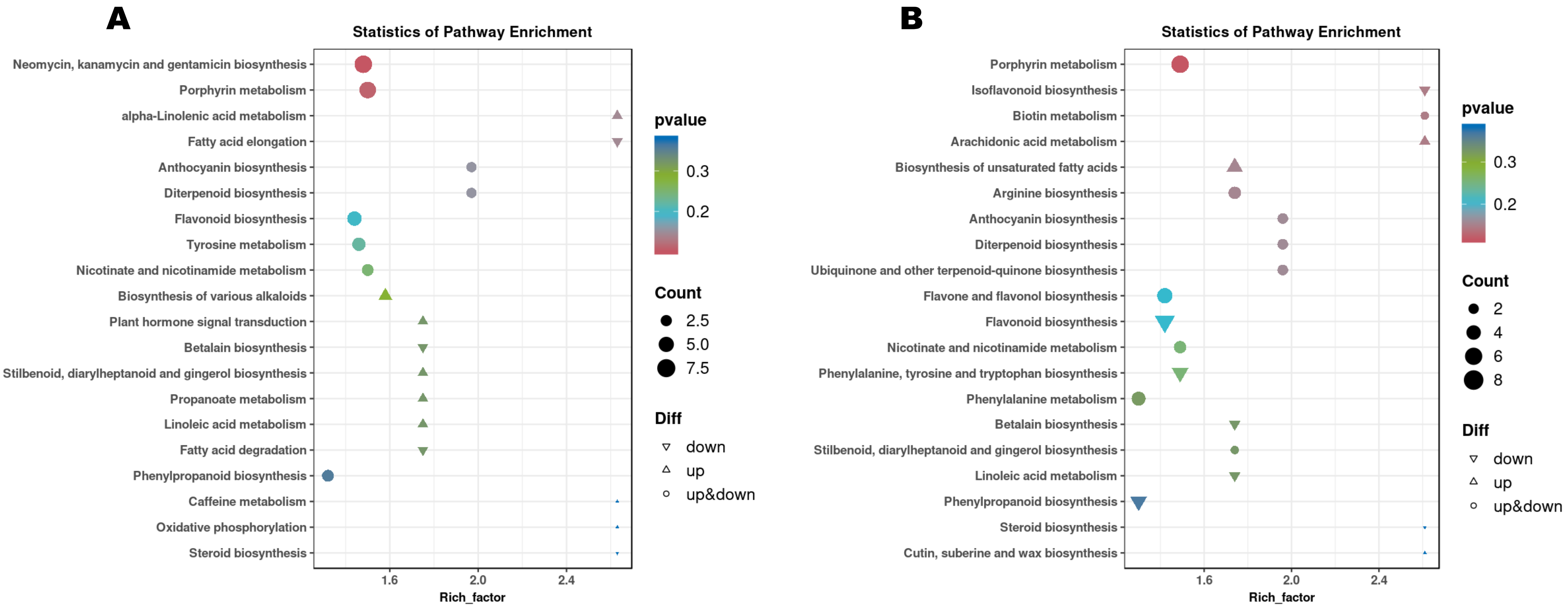

Figure S5. KEGG annotation and enrichment analysis of DMs between T3_vs_T4 (A) and T4_vs_T5 (B). T1, T2, T3, T4, and T5 indicate the vegetative stage, floral meristem transition stage, tepal primordia differentiation stage, stamen primordia differentiation stage, and pistil primordia differentiation stage, respectively.

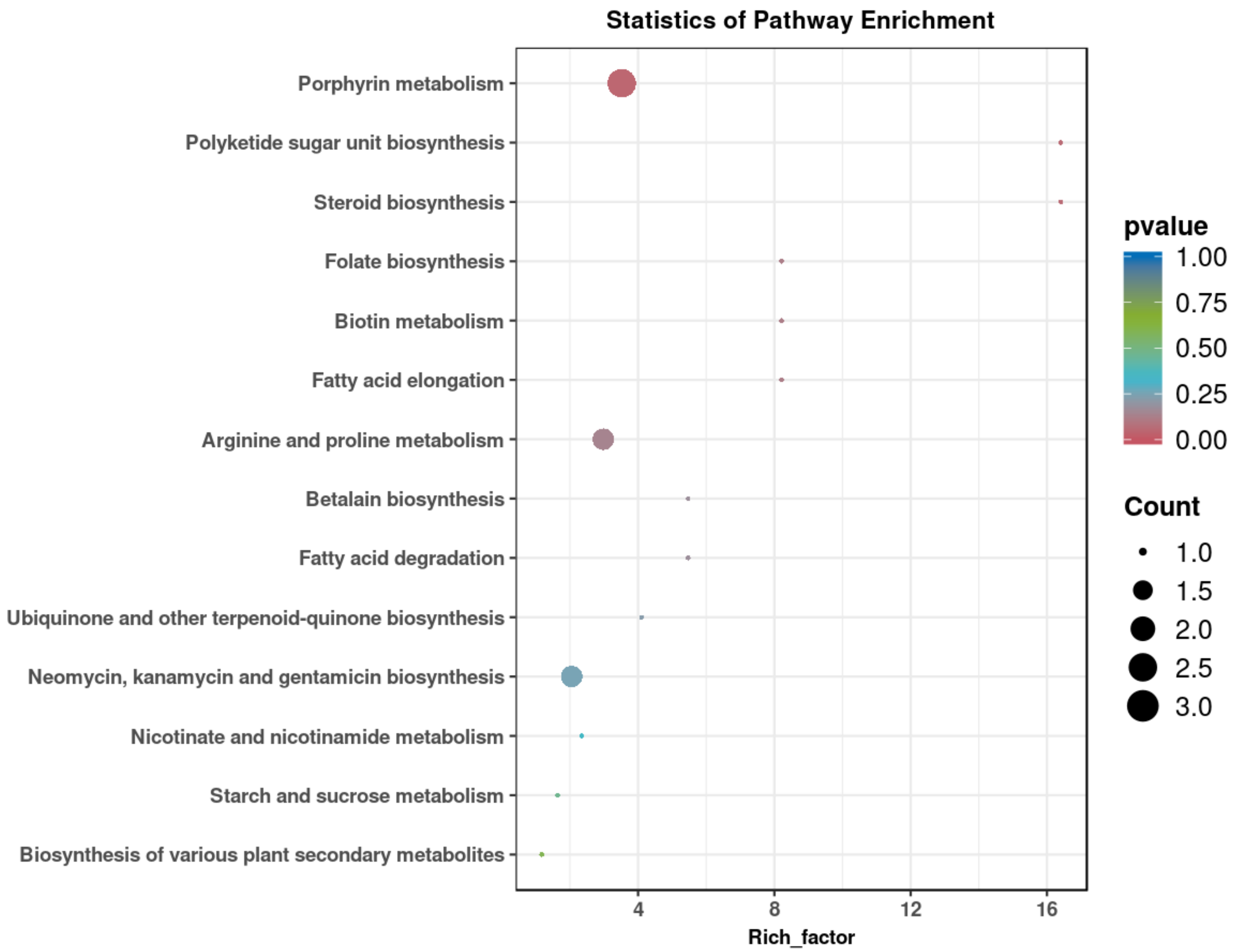

Figure S6. KEGG annotation and enrichment analysis of core overlapped DMs

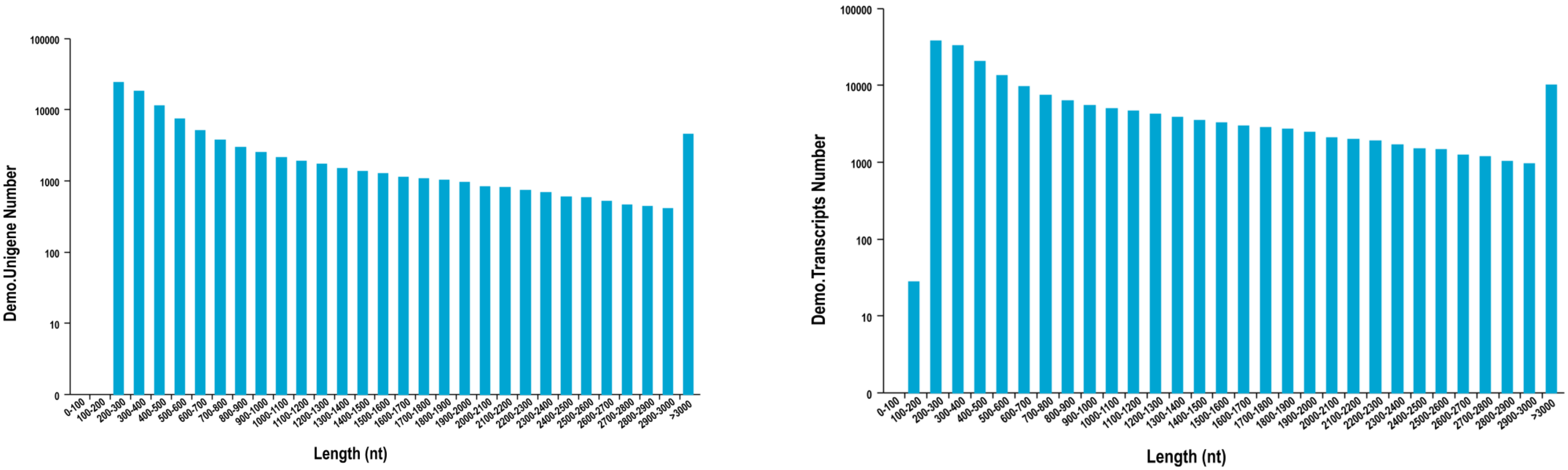

Figure S7. Distribution of the length of Unigenes (A) and transcripts (B).

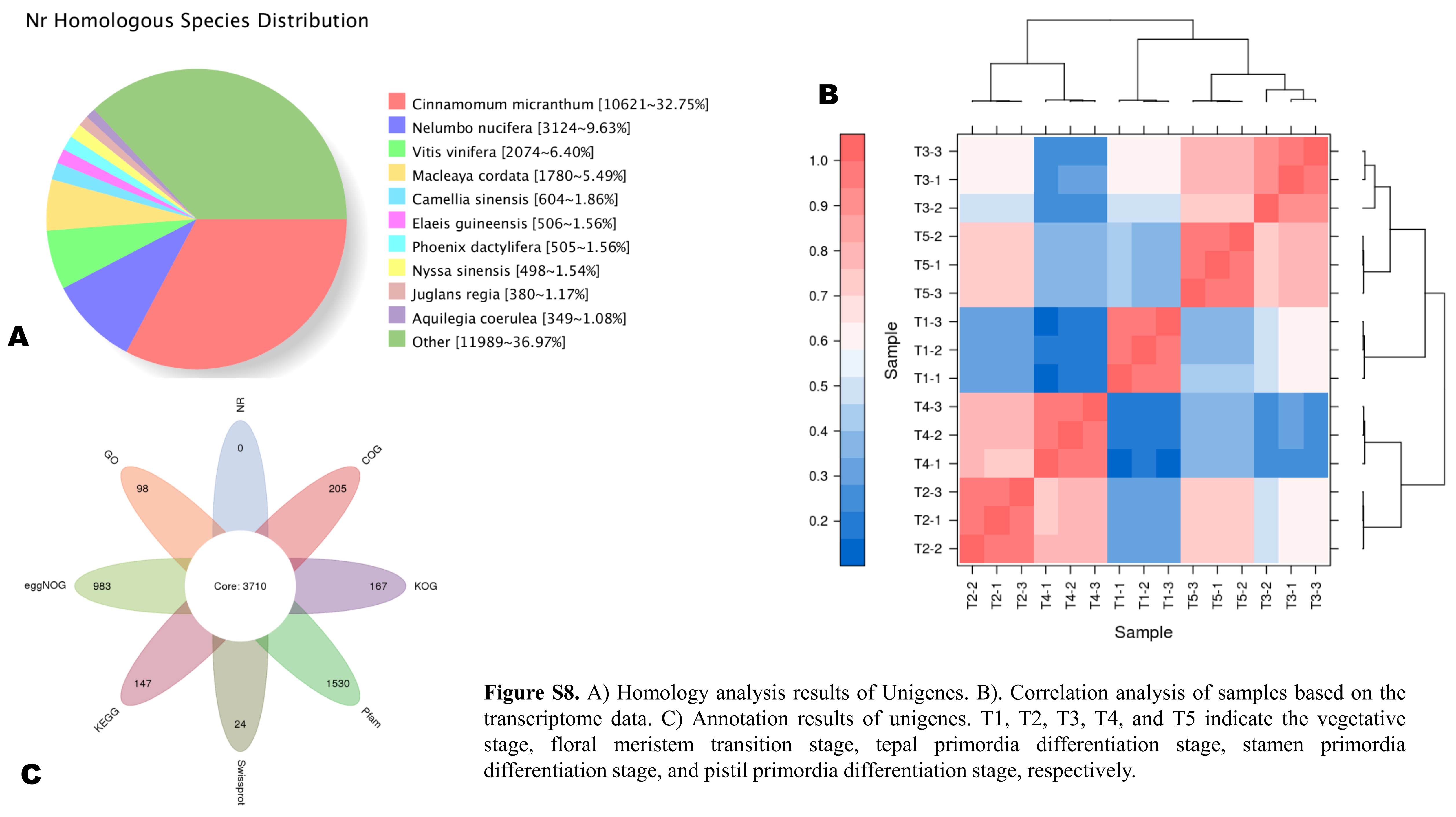

Figure S8. A) Homology analysis results of Unigenes. B). Correlation analysis of samples based on the transcriptome data. C) Annotation results of unigenes. T1, T2, T3, T4, and T5 indicate the vegetative stage, floral meristem transition stage, tepal primordia differentiation stage, stamen primordia differentiation stage, and pistil primordia differentiation stage, respectively.

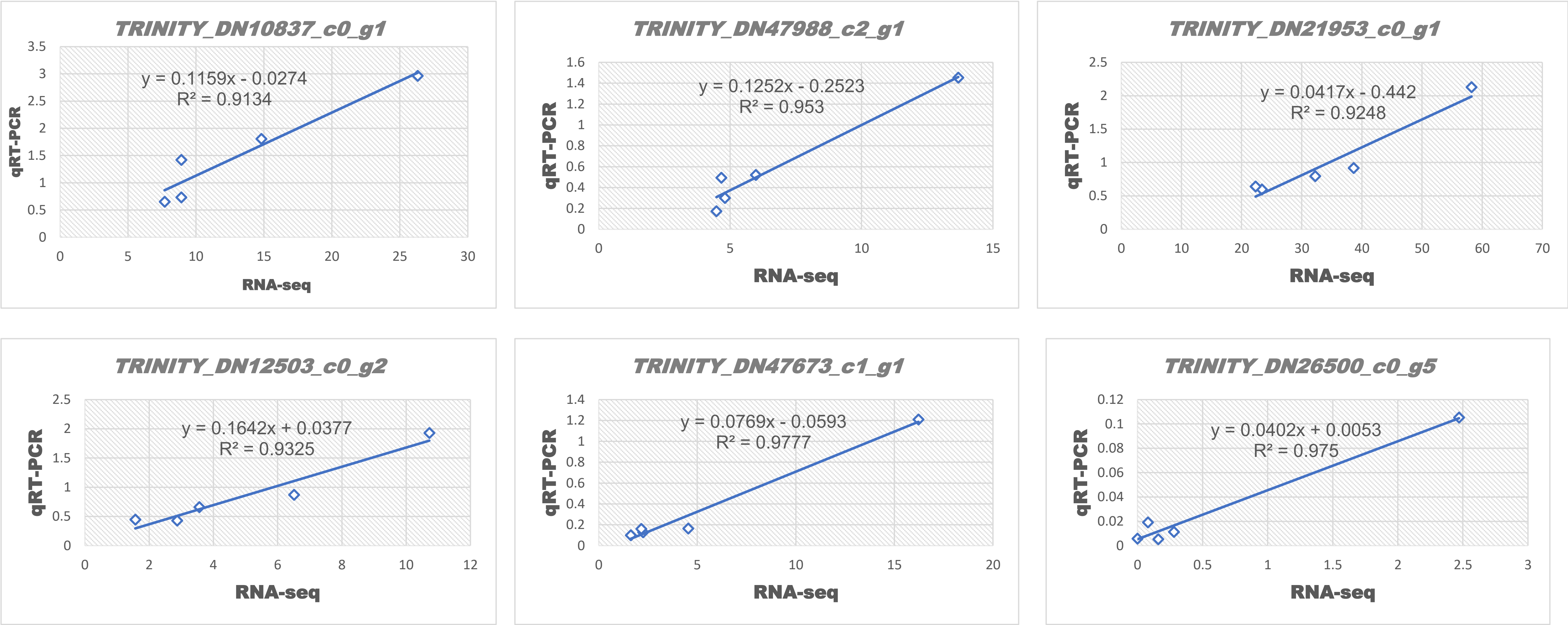

Figure S9. qRT-PRC validation of the RNA-seq data.

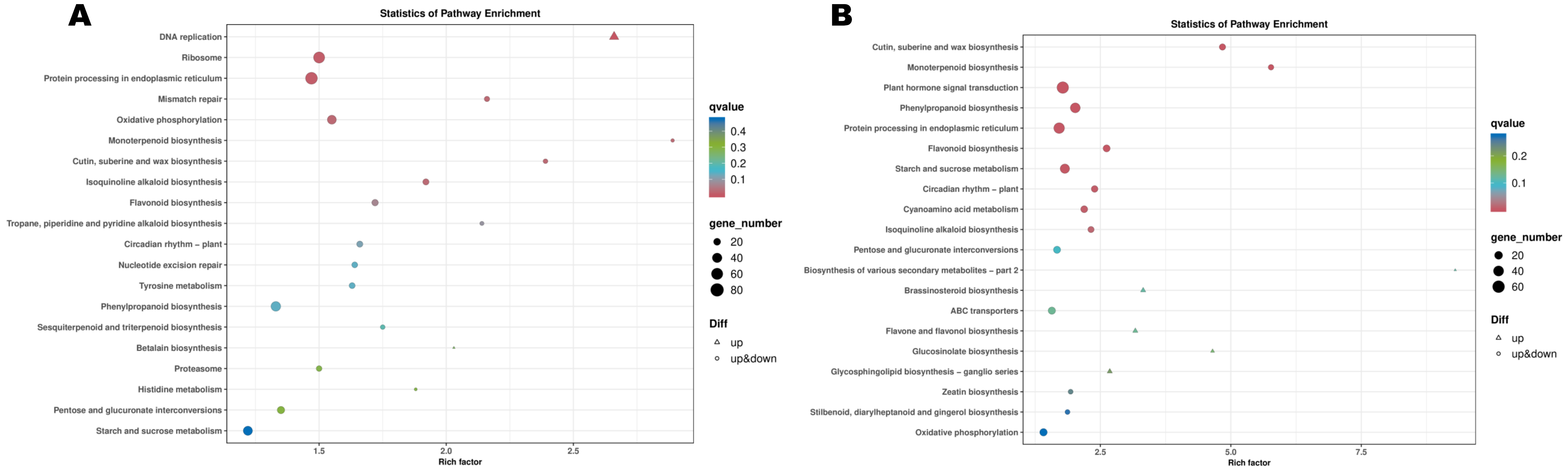

Figure S10. KEGG annotation and enrichment analysis of DEGs between T3_vs_T4 (A) and T4_vs_T5 (B). T1, T2, T3, T4, and T5 indicate the vegetative stage, floral meristem transition stage, tepal primordia differentiation stage, stamen primordia differentiation stage, and pistil primordia differentiation stage, respectively.

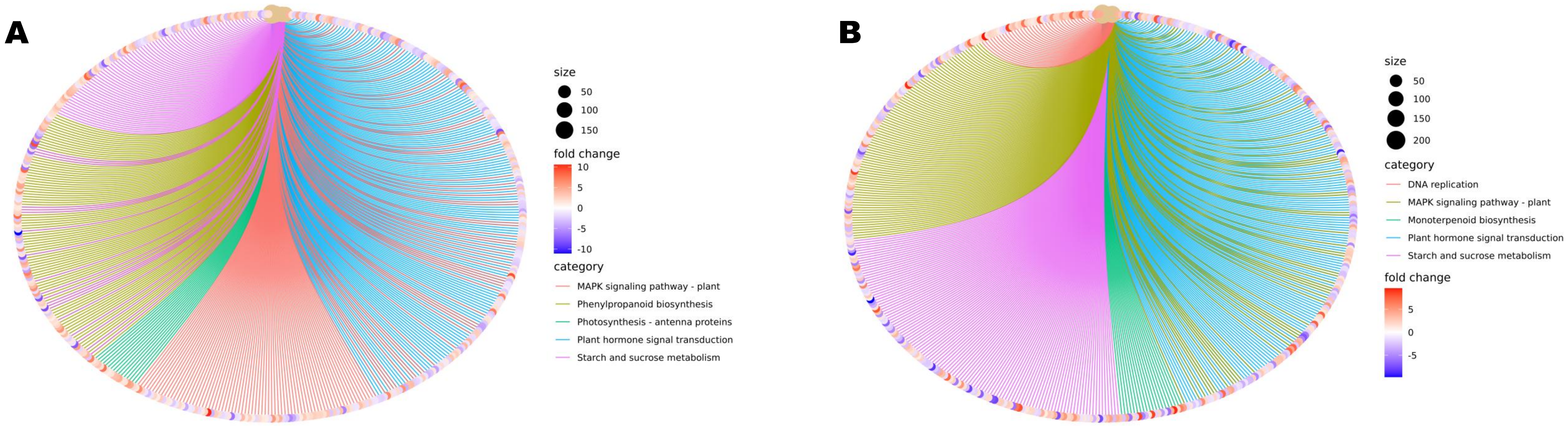

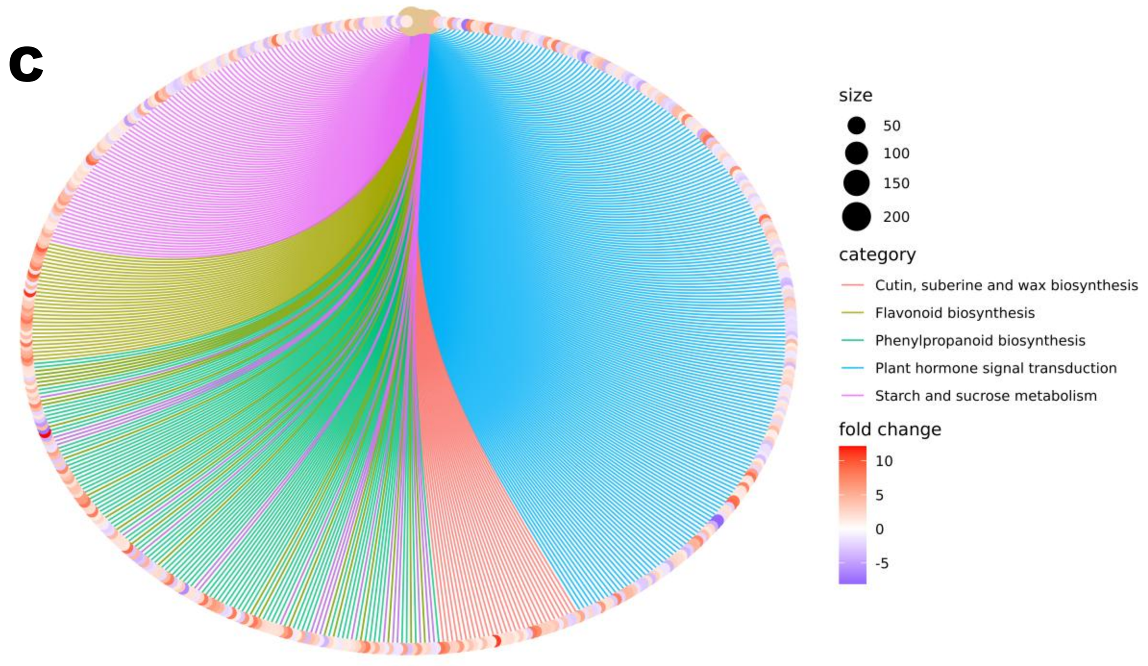

Figure S11. A- C) KEGG annotation and enrichment analysis of DEGs between T1_vs_T3, T1_vs_T4, T1_vs_T5, respectively. T1, T2, T3, T4, and T5 indicate the vegetative stage, floral meristem transition stage, tepal primordia differentiation stage, stamen primordia differentiation stage, and pistil primordia differentiation stage, respectively.

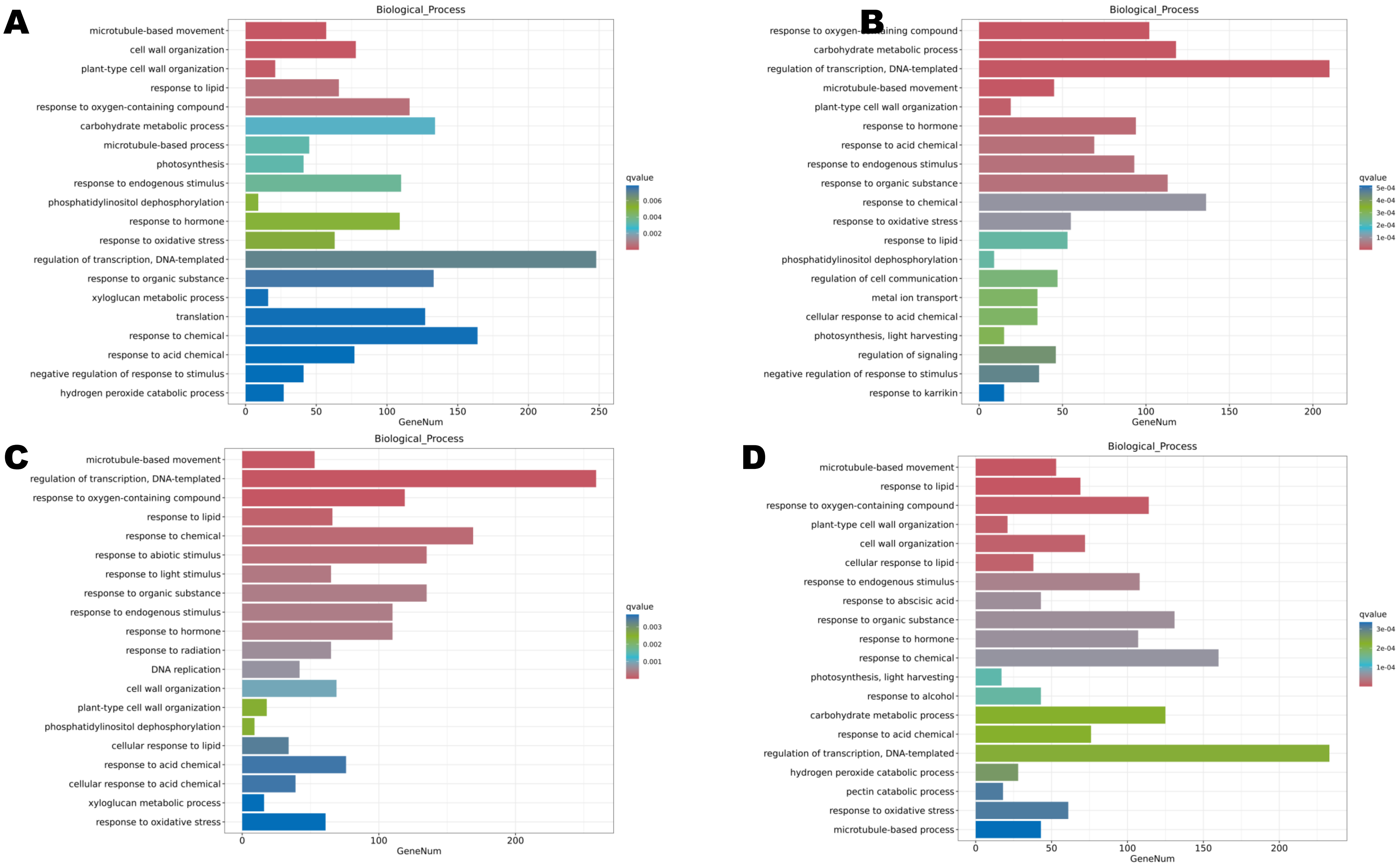

Figure S12. A- D) GO annotation and enrichment analysis of DEGs between T1_vs_T2, T1_vs_T3, T1_vs_T4, T1_vs_T5, respectively. T1, T2, T3, T4, and T5 indicate the vegetative stage, floral meristem transition stage, tepal primordia differentiation stage, stamen primordia differentiation stage, and pistil primordia differentiation stage, respectively.

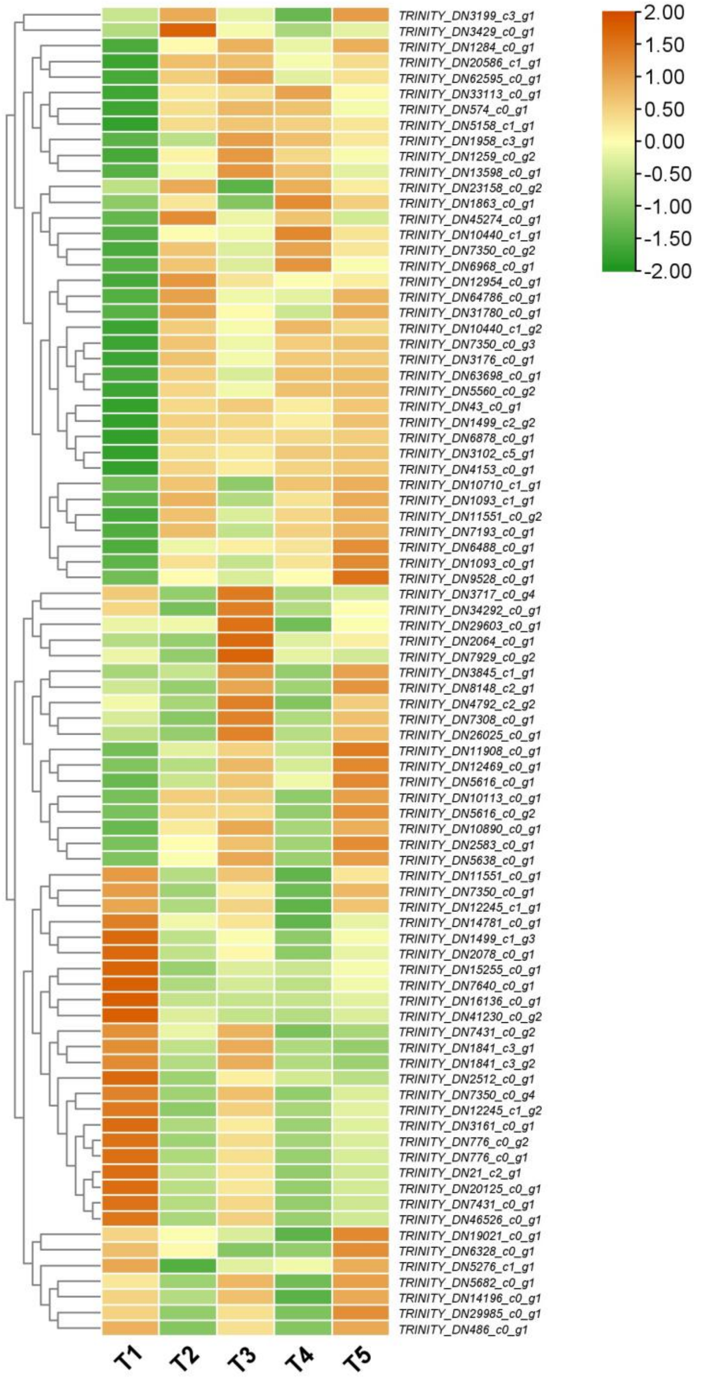

Figure S13. Expression patterns of auxin-related DEGs. T1, T2, T3, T4, and T5 indicate the vegetative stage, floral meristem transition stage, tepal primordia differentiation stage, stamen primordia differentiation stage, and pistil primordia differentiation stage, respectively.

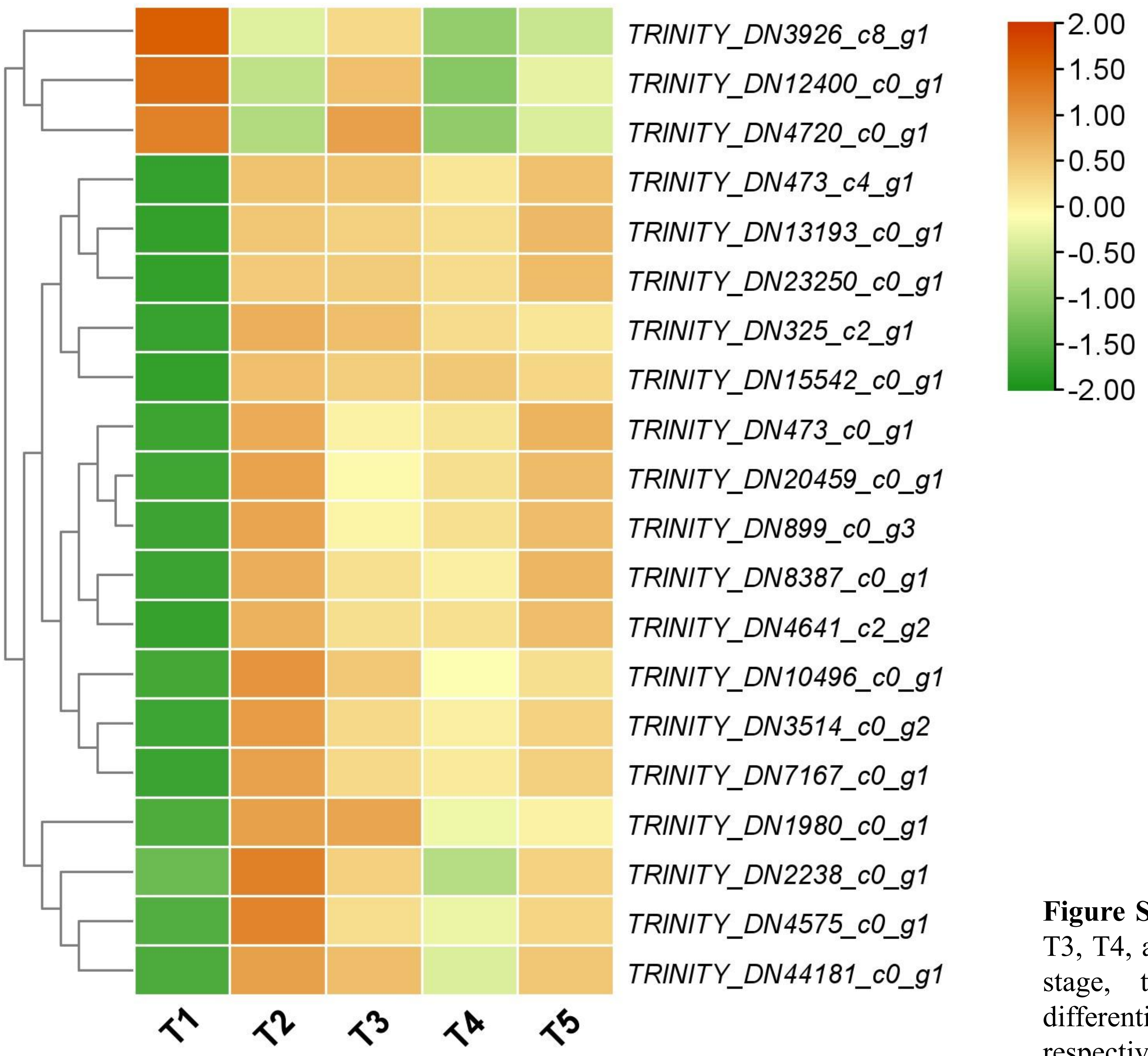

Figure S14. Expression patterns of Chlorophyll-related DEGs. T1, T2, T3, T4, and T5 indicate the vegetative stage, floral meristem transition stage, tepal primordia differentiation stage, stamen primordia differentiation stage, and pistil primordia differentiation stage, respectively.
